# Non-muscle myosin IIB (*Myh10*) is required for epicardial function and coronary vessel formation during mammalian development

**DOI:** 10.1371/journal.pgen.1007068

**Published:** 2017-10-30

**Authors:** Liam A. Ridge, Karen Mitchell, Ali Al-Anbaki, Wasay Mohiuddin Shaikh Qureshi, Louise A. Stephen, Gennadiy Tenin, Yinhui Lu, Irina-Elena Lupu, Christopher Clowes, Abigail Robertson, Emma Barnes, Jayne A. Wright, Bernard Keavney, Elisabeth Ehler, Simon C. Lovell, Karl E. Kadler, Kathryn E. Hentges

**Affiliations:** 1 Division of Evolution and Genome Sciences, School of Biological Sciences, Faculty of Biology, Medicine, and Health, Manchester Academic Health Science Centre, University of Manchester, Manchester, United Kingdom; 2 Wellcome Trust Centre for Cell-Matrix Research, Division of Cell-Matrix Biology and Regenerative Medicine, Faculty of Biology, Medicine, and Health, Manchester Academic Health Science Centre, University of Manchester, Manchester, United Kingdom; 3 Syngenta Ltd, Jealott’s Hill International Research Centre, Bracknell, United Kingdom; 4 Division of Cardiovascular Sciences, School of Medical Sciences, Faculty of Biology, Medicine, and Health, Manchester Academic Health Science Centre, University of Manchester, Manchester, United Kingdom; 5 Manchester Heart Centre, Central Manchester University Hospitals NHS Foundation Trust, Manchester, United Kingdom; 6 The Randall Division of Cell and Molecular Biophysics and the Cardiovascular Division, Kings College London, London, United Kingdom; The University of Chicago, UNITED STATES

## Abstract

The coronary vasculature is an essential vessel network providing the blood supply to the heart. Disruptions in coronary blood flow contribute to cardiac disease, a major cause of premature death worldwide. The generation of treatments for cardiovascular disease will be aided by a deeper understanding of the developmental processes that underpin coronary vessel formation. From an ENU mutagenesis screen, we have isolated a mouse mutant displaying embryonic hydrocephalus and cardiac defects (*EHC*). Positional cloning and candidate gene analysis revealed that the *EHC* phenotype results from a point mutation in a splice donor site of the *Myh10* gene, which encodes NMHC IIB. Complementation testing confirmed that the *Myh10* mutation causes the *EHC* phenotype. Characterisation of the *EHC* cardiac defects revealed abnormalities in myocardial development, consistent with observations from previously generated NMHC IIB null mouse lines. Analysis of the *EHC* mutant hearts also identified defects in the formation of the coronary vasculature. We attribute the coronary vessel abnormalities to defective epicardial cell function, as the *EHC* epicardium displays an abnormal cell morphology, reduced capacity to undergo epithelial-mesenchymal transition (EMT), and impaired migration of epicardial-derived cells (EPDCs) into the myocardium. Our studies on the *EHC* mutant demonstrate a requirement for NMHC IIB in epicardial function and coronary vessel formation, highlighting the importance of this protein in cardiac development and ultimately, embryonic survival.

## Author summary

In order for the heart to function properly it must have its own blood supply. Blood is delivered to the heart through a system of vessels called the coronary vasculature. During development, some of the cells that form the coronary vessels originate from the epicardium, the outer layer of the heart. These epicardial-derived cells migrate into the cardiac tissue where they contribute to the formation of the coronary vascular network. We have found that a mouse mutant containing a mutation in the gene *Myh10*, which encodes the cytoskeletal protein non-muscle myosin IIB, fails to form the coronary vasculature. Our work reveals defects in the epicardium, which contribute to the lack of coronary vessel development in this mutant. Surprisingly, we discovered that whilst the mutant epicardial cells are capable of movement when extracted from the embryo, these cells fail to mobilise into a vascular network in the context of the developing embryonic heart. We propose that this migration failure is due to abnormalities in the extracellular environment in the mutant heart. This work highlights the importance of the *Myh10* gene in the critical developmental process of coronary vessel formation.

## Introduction

A functional coronary vasculature is essential to supply the heart with oxygenated blood. Cessation of the coronary circulation deprives the working myocardium of oxygen and nutrients, leading to irreversible damage to cardiac muscle and myocardial infarction. Coronary artery disease is the main form of cardiovascular disease, and causes significant morbidity and mortality world-wide [1]. Although mammals and other higher vertebrates have insufficient capacity to restore cardiac function following ischemia, a number of studies that exogenously reactivate elements of embryonic coronary vessel formation have demonstrated neovascularisation and regeneration of the infarcted mouse heart, consequently improving cardiac function [2–5]. Moreover, experiments using thymosin β4 have revealed that the specific restoration of the quiescent adult epicardium, the outer epithelial layer of the heart, to an embryonic state, permits the activation of cardiac precursors that contribute to neovascularisation *in vitro* [6] and *in vivo* [2, 7]. However, our comprehension of both the cellular and molecular mechanisms that control this regeneration remain incomplete. Therefore, a deeper understanding of the processes that underpin coronary vessel formation may facilitate the generation of advanced and novel therapies to repair the injured heart.

During mammalian cardiac development cells from the proepicardial organ (PEO) migrate onto the surface of the heart and adhere to the nascent myocardium of the post-looped heart tube [8]. This gives rise to an outer epithelial layer, termed the epicardium, which completely envelops the developing heart. Epicardial function is critical for cardiac development, since the epicardium provides a source of paracrine signals for myocardial growth (reviewed in [9–11]). Additionally, as cardiac development progresses, a subset of epicardial cells undergo EMT and migrate through the subepicardial space to contribute to the formation of the coronary vasculature and cardiac fibroblasts (reviewed in [11–15]). While the contribution of the epicardium to the endothelial cell lining of the coronary vessels has been challenged recently [15, 16], it is clear that epicardial function is absolutely essential to establish the coronary vasculature and facilitate cardiogenesis. Furthermore, reactivating embryonic processes in the quiescent adult epicardium has been shown to facilitate the repair and regeneration of cardiac tissue in response to injury, highlighting the therapeutic potential of this tissue [5, 17, 18].

A comprehensive understanding of the critical molecular mechanisms that underpin epicardial function during mammalian cardiogenesis is needed to facilitate the development of epicardial-based therapies. To this end, we have studied mouse mutants with cardiac defects isolated from a balancer chromosome mutagenesis screen. We found that the *l11Jus27* mutant [19] carried two different embryonic lethal mutations, one of which displayed a phenotype of embryonic hydrocephalus and cardiac defects (*EHC*). *EHC* mutant embryos fail to form a mature, functional coronary system, resulting in late-gestation lethality. We identified a mutation in a splice-donor site in the *Myh10* gene as the cause of the *EHC* phenotype. *Myh10* encodes Non-Muscle Myosin Heavy Chain IIB (NMHC IIB), a component of the protein hexamer Non-Muscle Myosin IIB (NMIIB). There are 3 different NMHC II isoforms (namely IIA, IIB and IIC), each displaying specific cellular expression and functionality (reviewed extensively in [20, 21]). NMHC IIB is a cytoskeletal protein with diverse functions, including: cytokinesis [22, 23], regulation of cell shape [24], adhesion [25, 26] and migration [27, 28]. Prior studies of other mutants with abnormal coronary vessel development have shown that these defects arise primarily from epicardial cell dysfunction [29–32]. Accordingly, *EHC* mutant epicardial cells form an abnormal epithelial layer on the surface of the heart. In addition, the migration of *EHC* epicardial-derived cells into the underlying myocardium is impaired. *EHC* epicardial cells also show decreased expression of EMT markers within the epicardium, suggesting that NMHC IIB not only plays an important role in regulating EPDC migration, but also in promoting epicardial EMT. Ultimately, NMHC IIB may therefore function in multiple processes during coronary vessel formation and cardiogenesis, which could potentially be manipulated to repair and regenerate the heart in the context of cardiovascular disease.

## Results

### Isolation of the *EHC* mutant

**Fig 1:**
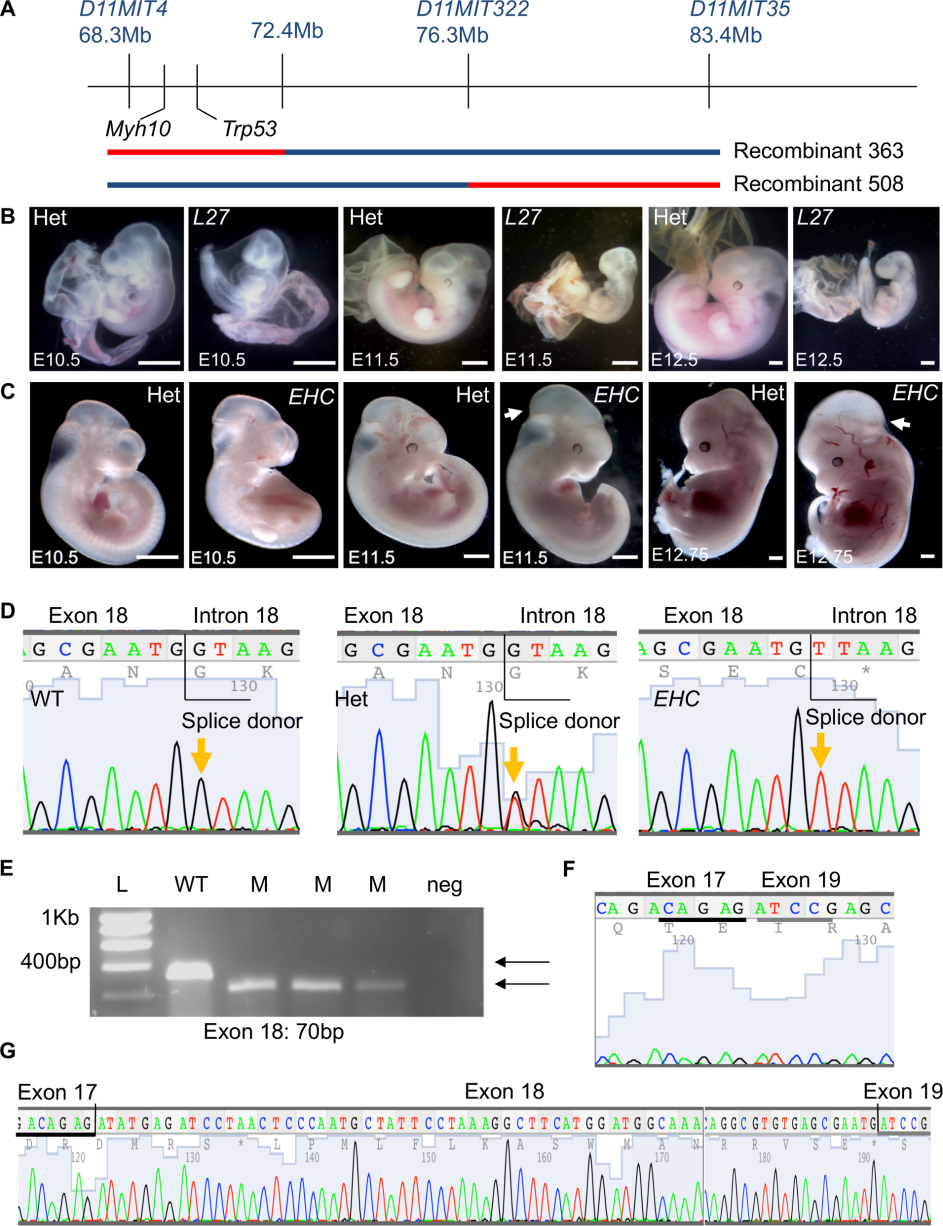
The *l11Jus27* mouse line carries two embryonic lethal mutations, one of which is in *Myh10*. A: Two recombinant mice show linkage of the phenotype (lethality) with distinct sub-regions of the mouse chromosome 11 balancer interval. The blue line indicates the 129S5 non-mutagenised chromosome. The C57BL/6 region (red) in recombinant mouse 363 extends beyond the balancer chromosome endpoint at *Trp53*. B: *l11Jus27* heterozygous and homozygous mutant embryos at E10.5, E11.5, and E12.5. For this and all subsequent figures, images are representative findings from a minimum of n = 3 observations unless otherwise stated. C: Dissections of embryos from crosses of *l11Jus27* heterozygotes with recombinant 363 reveal that at E10.5 –E12.75 the *l11Jus27* phenotype is not apparent in homozygous C57BL/6 embryos. At E11.5 and E12.75 embryos homozygous for C57BL/6 DNA have prominent hydrocephalus (arrow), revealing a second embryonic lethal phenotype in the *l11Jus27* line. D: Sequencing of the *Myh10* gene reveals a ‘G’ to ‘T’ point mutation in the splice donor site of exon 18 (orange arrows). E: The *Myh10* point mutation causes exon 18 to be skipped in the *Myh10* mutant transcript. RT-PCR in three homozygous *EHC* mutant embryos (M) reveals a smaller transcript than in one wild type (WT) embryo. The reduction in size of the PCR product is consistent with skipping exon 18. F: Sequencing *Myh10* transcript from mutant embryos confirms that exon 18 is missing, causing exons 17 and 19 to be joined. G: A wild type transcript that contains exons 17, 18, and 19. The black and grey lines in (F) and (G) highlight the sequence from exon 17 and sequence from exon 19 respectively shown in both the mutant and wild type. Scale bars: 1mm. Abbreviations: L27: *l11Jus27*; L: molecular size ladder; Het: heterozygote; WT: wild type; M: mutant; neg: negative control.

A study of ENU (*N*-ethyl-*N*-nitrosourea) mutagenised mouse strains with embryonic lethal recessive mutations revealed that homozygous mutant embryos from the *l11Jus27* mouse line displayed enlarged, distended hearts with oedema [19]. A meiotic mapping approach was employed to further refine the *l11Jus27* candidate region. Animals with recombination events within the balancer chromosome interval were test-crossed to known *l11Jus27* carriers, and viable offspring were genotyped for several markers in the balancer chromosome region. Correlations between the inheritance of C57BL/6 genomic DNA (mutagenised strain) and the *l11Jus27* phenotype (early embryonic lethality) were evaluated. The failure of a recombinant animal crossed to a known *l11Jus27* carrier to produce homozygous C57BL/6 offspring would suggest that the homozygous C57BL/6 embryos died *in utero*, consistent with the *l11Jus27* phenotype. This finding would indicate that the *l11Jus27* mutation is located in the genomic region where the recombinant animal has inherited C57BL/6 DNA. Unexpectedly, two recombinant mice failed to produce homozygous C57BL/6 viable offspring when mated to known *l11Jus27* heterozygotes (Fig 1A). Both of these animals carried C57BL/6 DNA in non-overlapping sub-regions of the balancer interval, suggesting that they could not carry the same embryonic lethal mutation. Recombinant 363 carried C57BL/6 genomic DNA in a 4.1Mb region extending beyond the *Trp53* endpoint of the balancer chromosome, and recombinant 508 carried C57BL/6 genomic DNA in the central region of the balancer interval (Fig 1A). To determine if recombinants 363 or 508 produced mutant embryos with the *l11Jus27* phenotype, timed matings were performed, and resulting embryos analysed at embryonic day (E) 10.5–12.5. At these developmental stages, the *l11Jus27* phenotype was apparent, with most mutant embryos dying by E12.5 (Fig 1B). However, homozygous C57BL/6 embryos produced from the cross of recombinant 363 to *l11Jus27* heterozygotes did not display the *l11Jus27* phenotype (Fig 1C). Instead, mutant embryos were viable past mid-gestation, and exhibited severe hydrocephalus in the mid-brain region from E11.5 (Fig 1C, arrows). We found that embryos generated from the cross of recombinant 508 did display the *l11Jus27* phenotype (Fig 1B), indicating that the *l11Jus27* mutation is located in the region between the polymorphic markers *D11MIT322* and *D11MIT35* on mouse chromosome 11.

Based on the new phenotype displayed in the offspring from recombinant mouse 363, we concluded that two embryonic lethal mutations were present in the *l11Jus27* mice, and that mutant embryos carrying both mutations exhibited the more severe *l11Jus27* phenotype. A new line of mice displaying the hydrocephalus phenotype was generated from crossing recombinant 363 to balancer chromosome animals, so that the new mutation could be maintained *in trans* to the balancer. This new mutant line was named *EHC*. Candidate gene analysis of the C57BL/6 region inherited by *EHC* mice revealed *Myh10* as a strong candidate gene. *Myh10* encodes NMHC IIB, the heavy chain component of the NMIIB protein complex, and targeted deletion of *Myh10* results in late-gestation lethality with hydrocephalus and cardiac defects [26, 33]. Therefore, due to similarities in phenotype, we sequenced *Myh10* genomic DNA from *EHC* mutant mice. We found a ‘G’ to ‘T’ transversion mutation in the splice donor site following exon 18 (Fig 1D). This mutation causes exon 18 to be skipped from the *Myh10 EHC* mutant transcript, producing a smaller product from an RT-PCR reaction performed with primers in exons 17 and 19 (Fig 1E). An abnormal *Myh10* transcript created from the fusion of exon 17 and exon 19 was present in *EHC* mutant embryos (Fig 1F), while wild type embryos contained a transcript with exons 17, 18, and 19 (Fig 1G).

**Fig 2:**
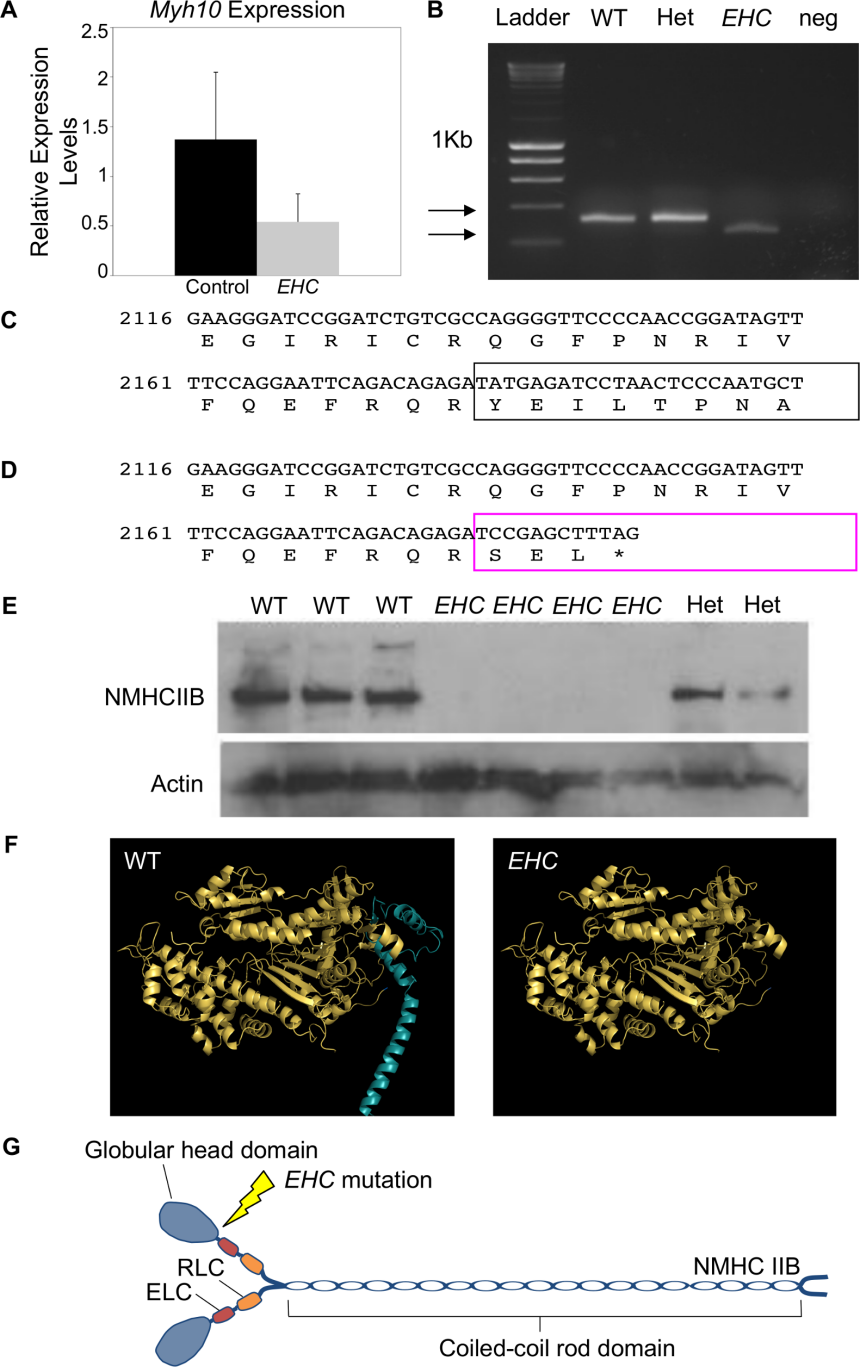
Predicted alterations to NMHC IIB protein in *EHC* mutants. A: Comparison of full-length *Myh10* transcript levels in control (WT or het) and mutant embryos by qPCR. B: RT-PCR amplification products from the region encompassing *Myh10* exon 17–19 in wild type, het, and mutant samples. C: *Myh10* wild type transcript and protein sequence is shown for exons 17 and 18. The boxed area highlights the translation of exon 18. Numbering refers to nucleotide positions of the *Myh10* transcript, with the initiation codon ‘A’ considered base 1. D: Predicted protein sequence for the *EHC* mutant transcript. The boxed area highlights the abnormal reading frame created by the fusion of exons 17 and 19, indicating that the mutant protein is truncated. E: Western blot with a C-terminal NMHC IIB antibody reveals that *EHC* mutant embryos do not produce full-length non-muscle myosin IIB. Actin is used as a loading control. F: Structural model of wild type NMHC IIB protein displays the head domain (left). The region deleted in the *EHC* mutant is shown in blue. The NMHC IIB protein predicted to be generated from the *Myh10* mutant transcript in *EHC* mutants (right). G: Schematic representation of wild type NMHC IIB dimers in complex with the essential light chains (ELC) and regulatory light chains (RLC) to form mature non-muscle myosin IIB. The location of the *EHC* mutation in the globular head domain of NMHC IIB is illustrated.

*EHC* mutant embryos showed a reduction in full-length *Myh10* transcript levels as analysed by qPCR (Fig 2A) or RT-PCR amplifying the region from exons 17–19 (Fig 2B). The NMHC IIB protein is a 230kDa molecule comprising 1976 amino acids [20, 21, 34]. The wild type NMHC IIB protein sequence translated from exons 17 and 18 is shown in Fig 2C. The abnormal fusion of exons 17 and 19 in the *EHC Myh10* transcript causes a change in the reading frame, resulting in a truncated protein (1–703 amino acids). Additionally, the final three amino acids are divergent from the wild type sequence with a YEI to SEL change (Fig 2D, boxed area). Using a C-terminal NMHC IIB antibody for western blotting, we confirmed that full-length NMHC IIB protein is not detectable in *EHC* mutant embryos (Fig 2E). Modelling the effect of the *EHC* mutation on the NMHC IIB protein demonstrated that the premature stop-codon will cause truncation of the protein in the actin binding head domain, resulting in the synthesis of NMHC IIB devoid of the coiled coil rod domain (Fig 2F). NMIIB is dependent upon interactions between the heavy chain rod domains to associate into bipolar filaments in order to exert its cellular function (Fig 2G) [20, 21]. We predict that if a truncated NMIIB protein was produced in *EHC* mutant embryos, this aberrant protein lacking the essential rod domain would be unable to partake in molecular interactions and therefore be unable to exert contractile force upon the actin cytoskeleton.

### Confirmation of the *EHC* causative mutation by complementation test

To confirm that the *EHC* causative mutation had been correctly mapped to *Myh10*, and subsequently caused loss of NMHC IIB function, we performed a complementation assay with a known *Myh10* null allele, denoted as *Myh10*∆. The *Myh10*∆ allele has a deletion of *Myh10* exon 2 [33], and does not synthesise full-length NMHC IIB protein (S1A Fig). As with the *EHC* mutants, homozygous *Myh10*∆ mutant embryos demonstrated late gestation embryonic lethality and were not present at the expected Mendelian ratios at birth (S1B Fig, Chi squared test p = 0.0035). Heterozygous *EHC* and *Myh10*∆ animals were intercrossed; the resultant progeny were genotyped and analysed for embryonic lethality and developmental defects. Analysis of Mendelian frequencies of *EHC*/*Myh10*∆ embryos at birth revealed a deviation from expectations due to late gestation embryonic lethality (S1C Fig, Chi squared test, p = 0.0278). These results indicated a failure of complementation between the two lines, and provided strong support for the hypothesis that the *Myh10* mutation causes the *EHC* phenotype by ablating NMHC IIB function.

### *EHC* mutants display embryonic hydrocephalus and cardiac defects

**Fig 3:**
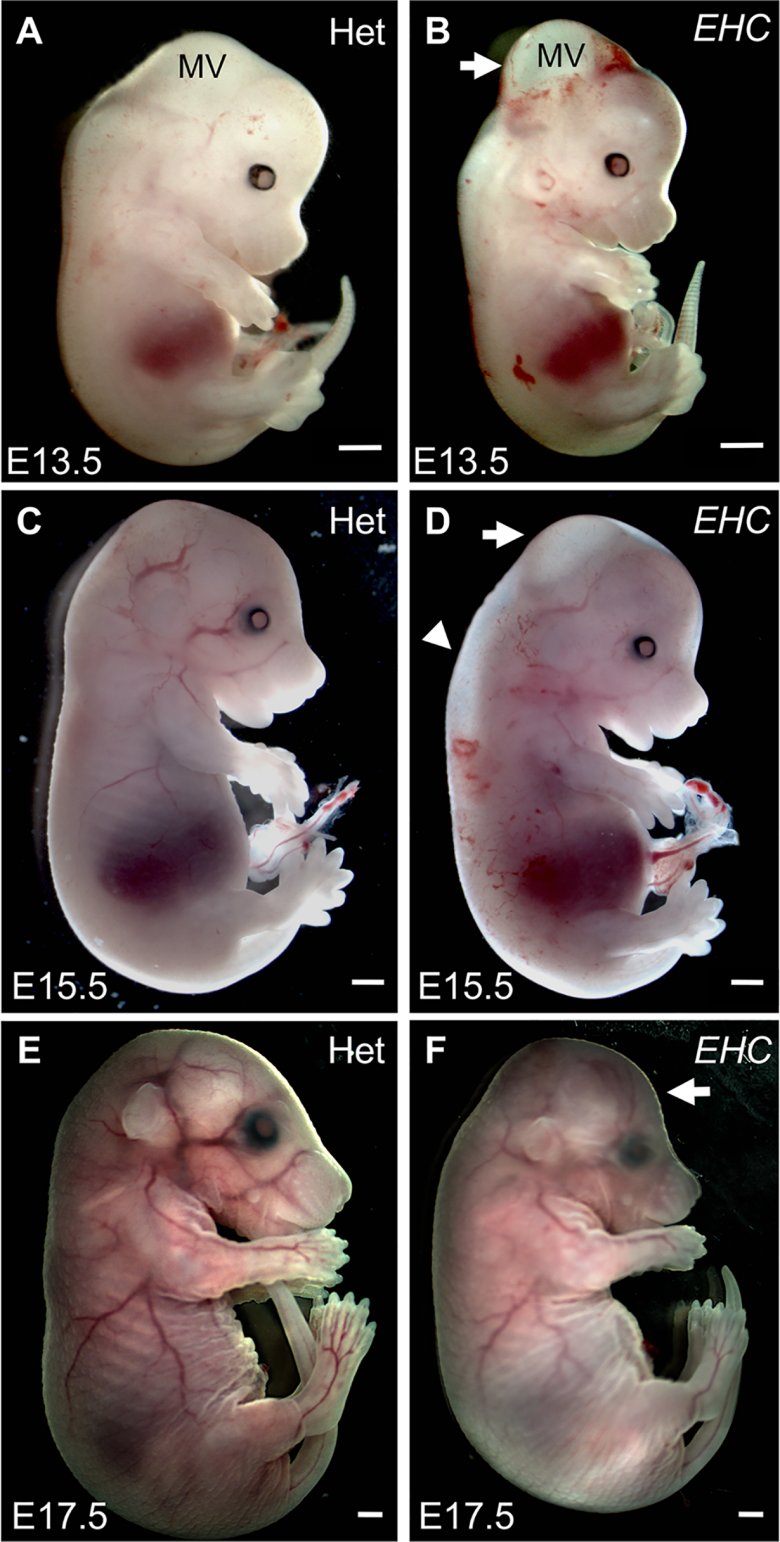
*EHC* mutant embryo morphology. A: Heterozygous littermate at E13.5. B: *EHC* mutant displays hydrocephalus in the mesencephalic vesicle (white arrow). C: Heterozygous littermate at E15.5. D: *EHC* mutant at E15.5 displays reduced hydrocephalus (arrow), although oedema in the spinal cord region is apparent (arrowhead). E: Heterozygous littermate at E17.5. F: *EHC* mutant has dome-shaped head (arrow), consistent with developmental defects arising from embryonic hydrocephalus. Scale bars: 1 mm. Abbreviations: MV: mesencephalic vesicle.

We concluded that *EHC* mutant embryos were alive at E12.5 since a regular heartbeat was observed at the time of dissection. We therefore examined the phenotype of *EHC* mutant embryos at late gestation. At E13.5, there is still prominent hydrocephalus in the mesencephalic vesicle (Fig 3A and 3B). At E15.5, *EHC* mutant embryos often displayed oedema in the spinal cord region (Fig 3C and 3D, arrowhead), whilst excess fluid within the mesencephalic vesicle persists (Fig 3C and 3D, arrow). It was rare to recover *EHC* mutant embryos past E16.5, but the embryos that did survive had abnormal dome-shaped heads, consistent with developmental hydrocephalus (Fig 3E and 3F, arrow). Based on the reduced recovery of the *EHC* mutants from dissections after E16.5, we conclude that the *EHC* phenotype causes late-gestation embryonic lethality, accompanied by defects in cranial development due to severe hydrocephalus in the early embryo.

**Fig 4:**
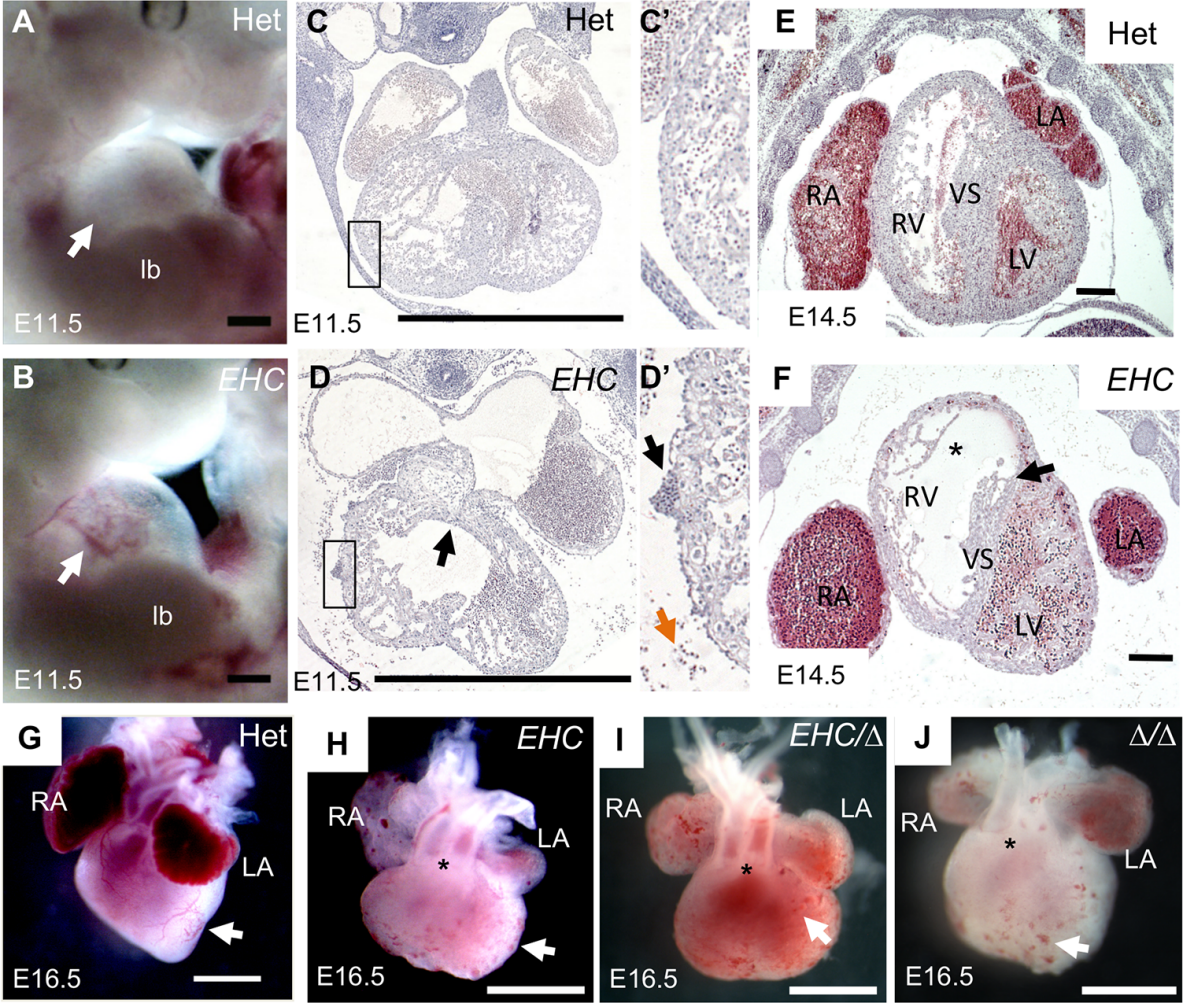
*EHC* mutants display defects in cardiac development. A: Heterozygous littermate control at E11.5 showing developing heart in pericardial sac. B: *EHC* mutant has pericardial effusion and blood visible in pericardial sac (arrow). C: Transverse section of control littermate at E11.5. The boxed region indicates the segment shown in panel C’. C’: Note the absence of blood cells on the cardiac surface and in the pericardial space. D: Transverse section of *EHC* mutant at E11.5 showing enlarged atria. The boxed region indicates the segment shown in panel D’. D’: Blood cells are present on the surface of the *EHC* heart (black arrow) and in the pericardial space (orange arrow). E: In heterozygous littermates at E14.5 the ventricular chamber is surrounded by a thick compact myocardium, which is highly organised. F: At E14.5, *EHC* mutants show a reduced compact ventricular myocardium (indicated by asterisk), accompanied by a thinning of the ventricular septal tissue (arrow). Note the abnormal atrial morphology and different localisation of the atria with respect to the ventricle compared to control (E). G: Heterozygous heart at E16.5 shows expected ventricular vascular connections and also coronary vessels on the cardiac surface (arrow). H: *EHC* mutant heart shows double-outlet right ventricle (DORV) (asterisk) as well as an absence of surface coronary vessels (arrow). Note the vesicle-like structures present on the ventricular surface. I: *EHC*/*Myh10*∆ and J: *Myh10*∆/*Myh10*∆ hearts at E16.5 similarly show DORV (asterisks) and ventricular vesicles (arrows). Scale bar = 200 μm (E, F), 1mm (A-D, G-J). Abbreviations: Het: heterozygote; lb: limb bud; LA: left atria, LV: left ventricle, RA: right atria, RV: right ventricle, VS: ventricular septum, ∆: *Myh10*∆.

As cardiac defects have been previously described for a targeted deletion of NMHC IIB [23, 33, 35], we examined cardiac development in the *EHC* mutant mouse. We found several similarities between the *EHC* cardiac phenotype and the defects described in the NMHC IIB knock out. First, upon dissection at E11.5 we observed pericardial effusion and blood in the pericardial sac of *EHC* mutant embryos (Fig 4B and 4D) and on the surface of the *EHC* mutant heart (Fig 4D’), phenotypes consistent with cardiac developmental defects. Membranous ventricular septal defects have been reported in NMHC IIB knock out animals [33, 35, 36]. However, we found that the endocardial cushions are present and have fused in *EHC* mutant embryos at E11.5 (Fig 4D, arrow), indicative of initial development of the septum. Later in gestation, *EHC* mutants at E14.5 have a thin ventricular septum with tearing in the membranous region, suggestive of a vulnerability to septal defects (Fig 4F, arrow). We also observed that *EHC* mutant ventricles display reduced trabeculation, a thinner compact myocardium, and disorganisation of cells in the ventricular myocardium (Fig 4D and 4F, asterisk). In addition, we detected defects in myocardial cytokinesis (S2A–S2D Fig), comparable to previously reported data from NMHC IIB knock out mice [23, 33, 37]. We observed double-outlet right ventricle (DORV), where the aorta erroneously stems from the right ventricle, in mutant embryos at E16.5 (Fig 4H asterisk), similar to findings from NMHC IIB knock out mice [33, 36]. Interestingly, DORV was not detected when NMHC IIB was specifically deleted in cardiomyocytes [35]. However, the DORV phenotype was completely penetrant in *EHC* hearts at E16.5. During our morphological inspection of E16.5 hearts, we also observed that mutant hearts displayed an abnormally rounded ventricular morphology, which lacked a prominent ventricular apex (Fig 4H–4J). In addition, *EHC* mutants displayed distended atria, abnormally positioned in relation to the ventricles (Fig 4F and 4H), and the ventricular surface was decorated with multiple blood filled vesicle-like structures (Fig 4D’ and 4H, arrows). These vesicles can be observed as early as E11.5 and persist until embryonic lethality. Again, these malformations were fully penetrant in mutant embryos by visual inspection at E16.5.

Additionally, the morphology of *EHC*/*Myh10*∆ and *Myh10*∆/*Myh10*∆ embryonic hearts closely resembles that of the *EHC* mutants (Fig 4I and 4J). Together, the phenotypic similarity of *EHC* and *Myh10*∆ homozygous mutants, combined with the failure of the *Myh10*∆ allele to complement the *EHC* allele, confirms that the *EHC* mutation causes complete loss of *Myh10* function, resulting in the observed *EHC* cardiac abnormalities.

### Mutant hearts lack coronary vasculature

**Fig 5:**
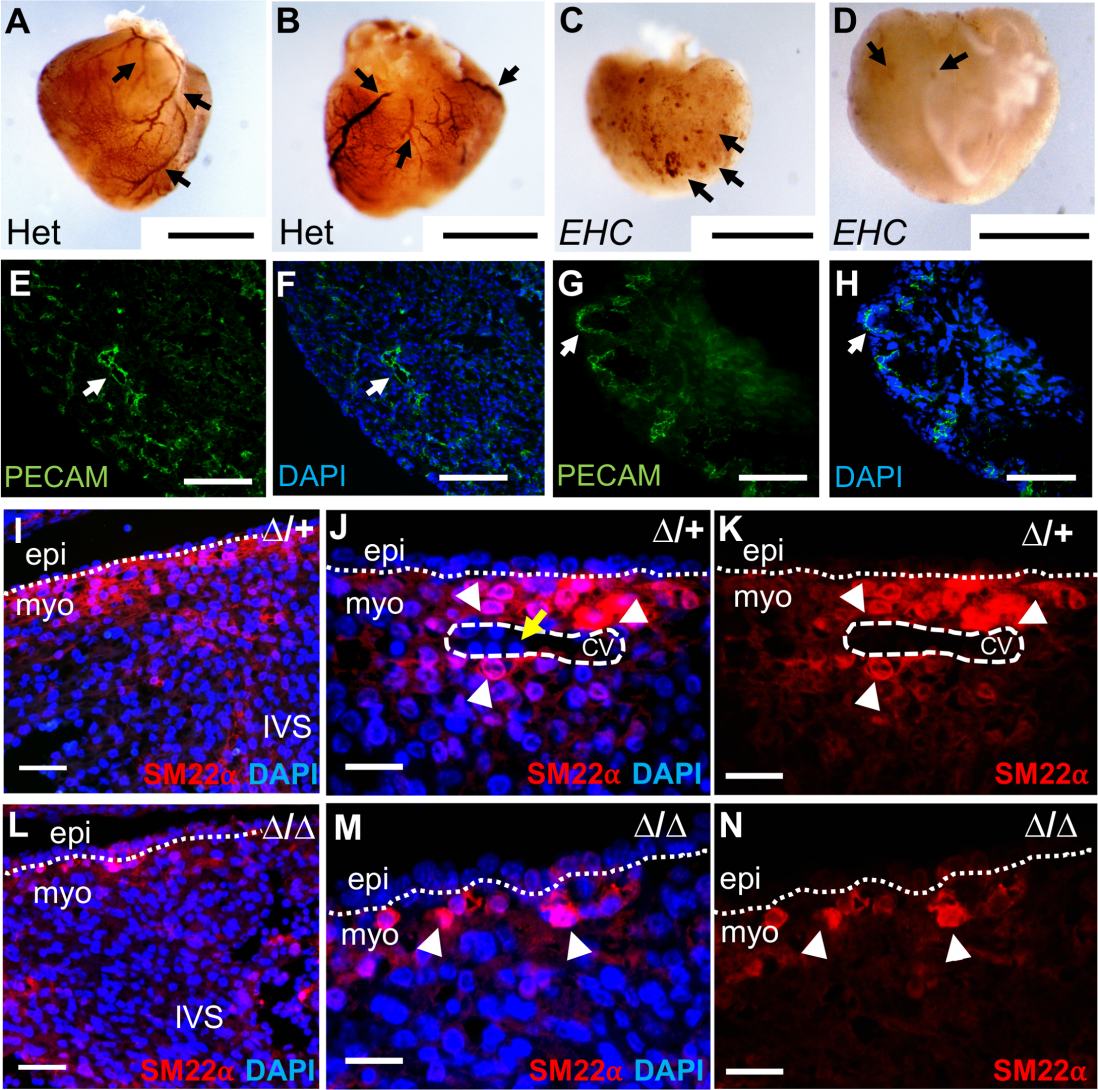
*EHC* mutants lack mature coronary vessels. PECAM-1 immunohistochemistry reveals prominent coronary vessels (arrows) on the ventral (A) and dorsal (B) surface of *EHC* heterozygous hearts. Only clusters of PECAM-1 expressing cells (arrows) are apparent on *EHC* homozygous mutant ventral (C) and dorsal (D) heart surface, with no clear evidence of coronary vessels. E-F: Section immunofluorescence for PECAM-1 (green) on *Myh10*∆ heterozygote at E14.5, demonstrating cells in myocardial region surrounding a developing vessel (arrow). G-H: Section immunofluorescence for PECAM-1 on *Myh10*∆ homozygous mutant at E14.5, demonstrating staining of clusters of cells on cardiac surface (arrow), consistent with whole mount staining in panels C-D. Staining for the smooth muscle cell marker SM22α reveals few smooth muscle cells within the interventricular septum region of the heterozygote (I) and *Myh10*∆ homozygous mutant (L). SM22α expressing cells are localised around a developing coronary vessel (white dashed circle) in heterozygous control hearts (J-K, arrowheads), and nucleated blood cells within the vessel can be visualised with DAPI (yellow arrow). No such structures are present in *Myh10*∆ homozygous mutant hearts, and smooth muscle cells expressing SM22α are less clustered in mutant hearts (M-N, arrowheads). Cell nuclei were labelled with DAPI (blue). Scale bars: A-D = 1 mm, E-H = 100 μm, I and L = 50 μm, J-K and M-N = 25 μm. Abbreviations: CV: coronary vessel, epi: epicardium, IVS: interventricular septum, myo: myocardium, PECAM: platelet endothelial cell adhesion molecule-1, ∆: *Myh10*∆.

It became strikingly apparent during our dissection observations that the *EHC* mutants lacked blood-filled coronary vessels on their ventricular surface, in contrast to heterozygous *EHC* control hearts (Fig 4G compared to 4H). The *Myh10*∆ homozygotes and *EHC*/*Myh10*∆ compound heterozygote mutant embryos also displayed a lack of coronary vessels (Fig 4I and 4J). To confirm that *EHC* and *Myh10*∆ mutant hearts lacked a mature coronary network, we performed immunohistochemical analysis for markers of cellular components of the coronary architecture, namely vascular endothelial cells (PECAM-1/CD31), and vascular smooth muscle cells (SM22α). Heterozygous littermates at E16.5 displayed clear PECAM-1 immunoreactivity, highlighting mature coronary vasculature in which vascular endothelial cells are organised into a large and extensively branched vascular network (Fig 5A and 5B arrows). In contrast, *EHC* mutants at E16.5 displayed only PECAM-1 immunoreactive surface cell clusters (Fig 5C and 5D arrows). A similar staining profile was observed in *EHC/Myh10∆* and *Myh10∆/Myh10∆* mutant hearts (S3 Fig). To further examine the extent of coronary defects, we evaluated the localisation of vascular endothelial and smooth muscle cells (vSMCs) at E14.5. In heterozygous *Myh10∆* controls, we observed intense PECAM-1 staining around the lumen of developing vessel structures, illustrating the presence of endothelial cells around the coronary vessels during maturation (Fig 5E and 5F arrow). However, *Myh10∆* homozygous mutant hearts displayed PECAM-1 staining surrounding clusters of blood cells on the cardiac surface (Fig 5G and 5H arrow), consistent with results from whole mount PECAM-1 staining of *EHC*, *EHC/Myh10∆* compound heterozygotes and *Myh10∆* homozygous mutant hearts (S3 Fig). Coronary endothelial cells are present in a capillary network on the surface of *EHC* mutant hearts, but lack organisation into larger vessels (S5J–S5K Fig). Smooth muscle cells were present in the heart of heterozygous control embryos (Fig 5I–5K), including surrounding coronary vessels (Fig 5J arrowheads). In the interventricular septum region, SM22α distribution in *Myh10∆* homozygous mutant hearts (Fig 5L) was similar to that of control hearts (Fig 5I). In the compact myocardium, *Myh10∆* homozygous mutants did not display organised clusters of smooth muscle cells or vascular structures (Fig 5M and 5N). Together, these experiments demonstrate that *EHC* and *Myh10∆* homozygous mutant hearts display similar defects in coronary vessel formation.

### NMHC IIB is not required in cardiomyocytes for coronary vessel development

**Fig 6:**
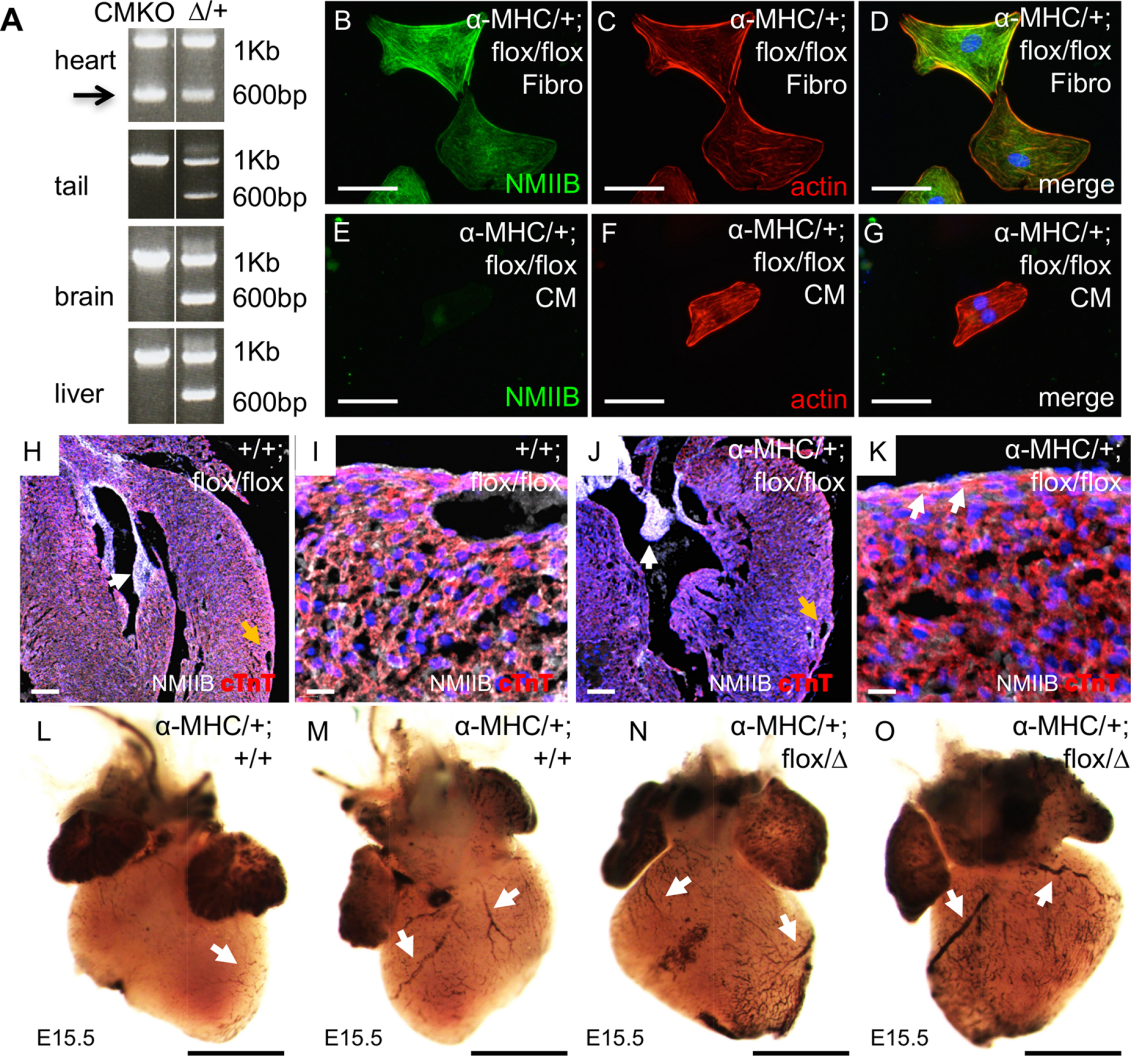
NMHC IIB is not required in cardiomyocytes for coronary vessel formation. A: Genomic PCR on tissues isolated from mice carrying the α*MHC-Cre* transgene and *Myh10 flox/+* alleles shows a PCR product of 600 bp in cardiac tissue, consistent with deletion of *Myh10* exon 2 from the genome (arrow). This product is not seen in tail, brain, or liver tissue. PCR results from a *Myh10∆/+* heterozygous animal are shown for the corresponding tissues, with the 600 bp product present in all tissues. The 1 Kb product represents the *Myh10* allele lacking exon 2 deletion. Products were sequenced to confirm specificity of PCR reactions. B: NMHC IIB protein (green) and C: actin (red; phalloidin stain) localisation in fibroblasts cultured from α*MHC-Cre; flox/flox* hearts. D: Merged image shows DAPI labeling of nuclei (blue). E: NMHC IIB protein (green) and F: actin (red; phalloidin stain) localisation in cardiomyocytes cultured from α*MHC-Cre; flox/flox* hearts. G: Merged image shows DAPI labelling of nuclei (blue). H: Expression patterns of NMHC IIB (white) and cardiac Troponin T (red), and nuclei stained with DAPI (blue) in α*MHC-Cre; Myh10+/+* control embryo with presence of coronary vessel (orange arrow). NMHC IIB expression is seen throughout heart including valve tissue (white arrow). Co-expression of NMHC IIB and cTnT appears pink. I: Higher magnification image of cardiac tissue from α*MHC-Cre; Myh10+/+* control embryo. J: Expression patterns of NMHC IIB (white) and cardiac Troponin T (red), and nuclei stained with DAPI (blue) in α*MHC-Cre; Myh10 flox/flox* control embryo with presence of coronary vessel (orange arrow). NMIIB expression is seen in valve tissue (white arrow) but reduced in myocardial region of heart, and is absent from cTnT positive cells, so that the pink staining indicating NMHC IIB and cTnT co-expression is reduced in the myocardium. K: Higher magnification image of cardiac tissue from α*MHC-Cre; Myh10 flox/flox* control embryo. Epicardial expression of NMHC IIB persists (white arrows). L: Ventral view of cardiac surface stained with DAB to identify blood cells in α*MHC-Cre; Myh10+/+* heart (arrow). M: Dorsal view of cardiac surface stained with DAB to identify blood cells in α*MHC-Cre; Myh10+/+* heart (arrows). N: Ventral view of cardiac surface stained with DAB to identify blood cells in α*MHC-Cre; Myh10 flox/∆* heart (arrows). O: Dorsal view of cardiac surface stained with DAB to detect endogenous peroxidase activity from blood cells in α*MHC-Cre; Myh10+ flox/∆* heart (arrows). Blood cells within vessels are present on the cardiac surface of cardiomyocyte-specific *Myh10* mutant hearts. Similar results were seen for α*MHC-Cre; Myh10 flox/flox* hearts. Scale bars: B-G = 50 μm, H and J = 100 μm, I and K = 15 μm, L-O = 400 μm. Abbreviations: α*MHC-Cre*: *Tg(Myh6-cre)*^*2182Mds/J*^, CM: cardiomyocyte, cTnT: cardiac troponin T, Fibro: fibroblast, NMIIB: NMHC IIB, ∆: *Myh10*∆.

Prior research has demonstrated that mice with a cardiomyocyte-specific deletion of *Myh10* are viable [35], suggesting that coronary vessel development must not be severely compromised when NMHC IIB function has been lost from cardiomyocytes. To investigate the dependence of coronary vessel development on cardiomyocytes NMHC IIB activity, we implemented the previously described strategy [35] to delete *Myh10* exon 2 in cells expressing the cardiomyocyte-specific α-*Myosin Heavy Chain-Cre* (α*MHC-Cre*) transgene. Confirmation of the genomic deletion of *Myh10* exon 2 in cardiac cells, but not tail, brain, or liver cells was demonstrated by genomic PCR for primers surrounding *Myh10* exon 2 (Fig 6A). These primers generate a 1 Kb product when exon 2 is present in the genome, and a 600bp product after deletion of *Myh10* exon 2. The 600bp deletion product is visible only in heart tissue (Fig 6A, arrow). We further demonstrated the cardiomyocyte specificity of the deletion of *Myh10* by isolating cardiac cells, dissociating them in culture, and subjecting the cells to fibroblast or cardiomyocyte culture protocols [38, 39]. NMHC IIB protein persists in fibroblast cells, which show characteristic morphology (Fig 6B–6D), but not in cardiomyocytes (Fig 6E–6G), which also have distinctive morphology in culture. Histological sections of hearts at E18.5 from control and *Myh10* cardiomyocyte-specific knock out embryos were examined by immunofluorescence for NMHC IIB and cardiac troponin T expression (Fig 6H–6K). Embryos inheriting the α*MHC-Cre* transgene and homozygous *Myh10* floxed alleles had reduced NMHC IIB expression within the myocardium, although expression of NMHC IIB can be seen in non-cardiomyocyte cell populations such as the endocardial cells present in the valve leaflets (Fig 6J, white arrow). Analysis of vessel development in control embryos and cardiomyocyte knockouts reveals the presence of blood cells in organised vessels on the cardiac surface, visible directly in dissections at E18.5 (S4A–S4D Fig), with PECAM-1 staining at E16.5 (S4E and S4F Fig), and following DAB staining of blood cells within heart tissue at E15.5 (Fig 6L–6O). These results confirm that cardiomyocyte expression of NMHC IIB is not required for the development of coronary vessels.

### *EHC* mutants display abnormal epicardial cell morphology

**Fig 7:**
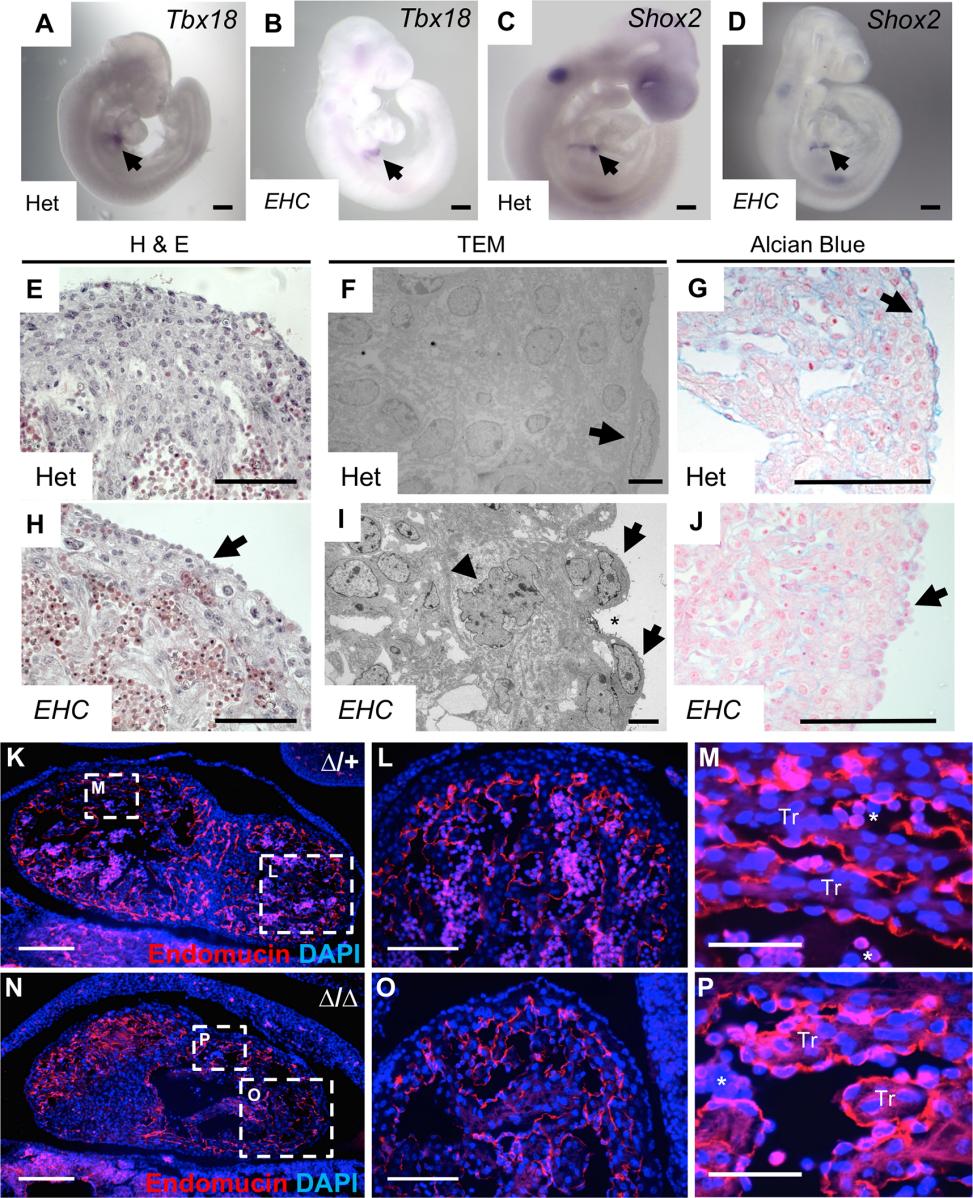
*EHC* mutants display epicardial defects. A: *In situ* hybridisation demonstrating *Tbx18* expression in the proepicardial organ of heterozygous and B: *EHC* homozygous mutant E9.5 embryos (arrows). C: *Shox2* expression is detected in the sinus venosus of heterozygous and D: *EHC* homozygous mutant E9.5 embryos (arrows). The expression patterns of *Tbx18* and *Shox2* are indistinguishable between controls and *EHC* mutants. E: Heterozygous embryos have an epithelial layer comprised of epicardial cells coating the developing ventricle at E14.5. F: Electron microscopy showing E16.5 epicardial cells from heterozygous mice having a flattened morphology (arrow) and forming a contiguous layer with adjacent cells. G: Alcian blue staining reveals a layer of glycosaminoglycans in the extracellular matrix between the epicardium and myocardium in heterozygotes at E11.5 (arrow). H: Epicardial cells are present on the surface of the *EHC* homozygous mutant myocardium (arrow) at E14.5. I: Mutant epicardial cells are irregular in shape and exhibit a rounded morphology (arrows), which generates gaps between neighbouring cells (asterisk). Subepicardial cells display evidence of disrupted cellular architecture maintenance (arrowhead). J: *EHC* mutants lack Alcian blue staining in the subepicardial tissue domain (arrow). K-M: Endomucin immunofluorescence staining (red) of control E11.5 coronal cardiac sections delineates the ventricular endocardium. N-P: Endomucin distribution in *Myh10∆* homozygous mutants is indistinguishable from controls. Areas shown at higher magnification in panels L-M and O-P are indicated with boxes on images K and N. Nucleated erythrocytes in panels M and P are indicated by asterisks. Scale bars: A-D = 250 μm, E and H = 60 μm, F and I = 5 μm, G and J = 125 μm, K and N = 200 μm, L and O = 100 μm, M and P = 50 μm. Abbreviations: H&E: haematoxylin and eosin, TEM: transmission electron micrograph Tr: trabeculae, ∆: *Myh10*∆.

Due to the evidence that cardiomyocyte NMHC IIB is not required for coronary vessel development, we sought to determine if defects in epicardial cell function may therefore underpin the *EHC* mutant phenotype. A number of seminal studies have demonstrated that the epicardium plays a crucial role in the formation of the coronary vessels during mammalian development [15, 40–45]. As the coronary vessels incorporate cells and signals from the epicardium and sinus venosus during their development [13, 15], we investigated if the specification of the epicardial precursor, the proepicardial organ, and the sinus venosus occurred correctly in *EHC* mutant embryos. *In situ* hybridisation for *Tbx18*, a proepicardial marker [46], and *Shox2*, which is expressed in the sinus venosus [47, 48], showed that these molecular markers are expressed in a similar pattern in heterozygous and *EHC* mutant developing embryos (Fig 7A–7D, arrows), suggesting that these coronary vessel precursor tissues were present during early development in mutant embryos.

Histology and transmission electron microscopy revealed that at E14.5, heterozygous hearts showed the formation of a characteristic epithelial layer on the surface of the myocardium (Fig 7E–7G) with flattened epicardial cells (Fig 7F, arrow). Although epicardial cells were clearly evident on the surface of the *EHC* mutant heart (Fig 7H, arrow), these cells had an unusual morphology compared to controls (Fig 7I, arrows). In addition, the *EHC* epicardium did not form a contiguous epithelial layer, and individual epicardial cells did not appear to form appropriate contacts with adjacent cells (Fig 7I, asterisk). The formation and maintenance of epicardial cell-cell gap junctions has previously been shown to be essential for correct epicardial cell function [49]. Interestingly, the ultrastructure of the subepicardial cell nucleus displayed an abnormal, multi-folded morphology, indicating defects in the maintenance of correct cellular architecture (Fig 7I, arrowhead). We calculated the percentage of epicardial cells with visually abnormal morphology in control and *EHC* mutant EM images, finding a statistically significant increase in the percentage of abnormal cells in *EHC* mutants (S2E–S2G Fig; Fisher’s exact test p<0.001).

The epicardium deposits extracellular matrix into the subepicardial space, which separates it from the underlying myocardium and is thought to play a critical role in the molecular communication between these tissues during embryonic development, homeostasis, and response to injury [13, 50, 51]. To evaluate possible defects in epicardial ECM deposition in the *EHC* mutants, we next analysed the subepicardial ECM by staining E11.5 cardiac sections with Alcian blue, a marker of glycosaminoglycans (GAGs) (Fig 7G and 7J). Prominent Alcian blue staining clearly delineated the subepicardial ECM in control hearts (Fig 7G, arrow). In contrast, *EHC* mutants lacked Alcian blue staining at the boundary between the epicardium and myocardium (Fig 7J, arrow), suggestive of abnormalities in the subepicardial ECM. Both mutant and control embryos display similar Alcian blue staining in the endocardial cushion mesenchyme (S2H–S2K Fig), illustrating that the localisation profile of GAGs within the heart is not universally disrupted in *EHC* mutants.

To evaluate potential requirements for NMHC IIB in other cardiac cell types with a role in coronary vessel development, we investigated whether or not NMIIB ablation disrupted the development of the endocardium by analysing the localisation of the endocardial marker endomucin, in both heterozygous control and *Myh10*∆ homozygous mutants E11.5 hearts (Fig 7K–7P). *Myh10*∆ homozygous mutants were utilised in these experiments to facilitate genotyping, as the loss of *Myh10* exon 2 can be determined from a single PCR rather than requiring genomic sequencing as needed to detect the *EHC* point mutation. No aberrations in endomucin staining were detected in *Myh10*∆ homozygous mutant embryos (Fig 7N–7P), suggesting that the formation of the endocardium is not dependent upon NMIIB function. Moreover, the developing atrioventricular valves, derived from endocardial tissue, are present in heterozygous control and *EHC* mutant embryos at E14.5 and E16.5 (S5A–S5D Fig). As the formation of cardiac valve structures is highly dependent upon a functional endocardium [13, 52], the presence of these structures in *EHC* mutants supports the hypothesis that endocardial function is not significantly impaired following loss of NMHC IIB. A recently published complementary study has demonstrated that coronary vessel development is impaired when NMHC IIB is deleted specifically within the epicardium [53], supporting our findings that epicardial abnormalities in *EHC* mutant mice contribute to defective coronary vessel development.

### NMHC IIB is the predominant form of NMII in the embryonic heart and epicardium

**Fig 8:**
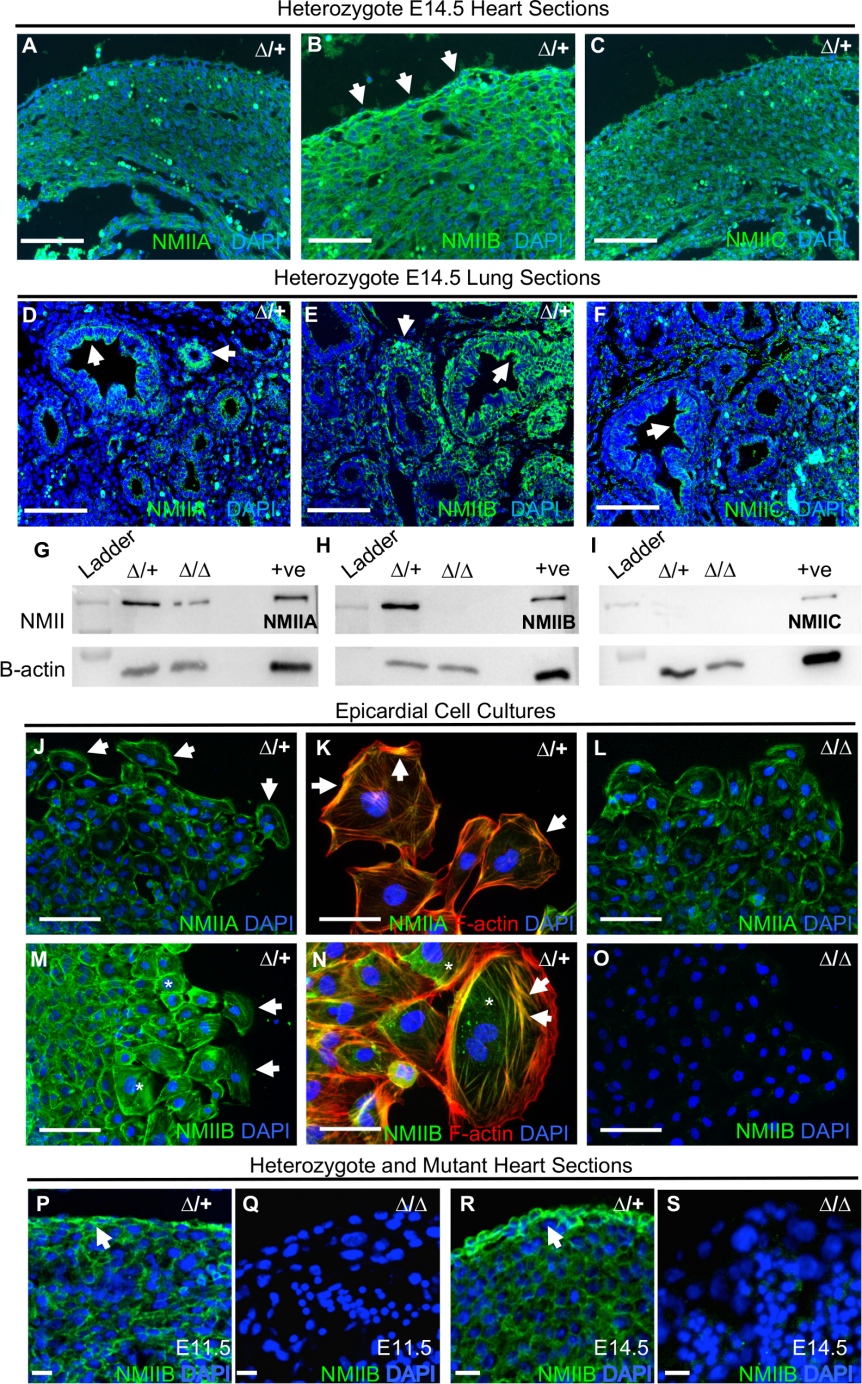
NMHC IIB is the predominant form of NMII in the embryonic heart and epicardium. A-C: Immunohistochemical comparison of the three different NMHC II isoforms (green) in the *Myh10*∆/+ E14.5 heart. NMHC IIB is the predominant isoform, and shows intense staining in the epicardium (B, arrows). D-F: E14.5 embryonic lung tissue was used as a control for the NMII isoform immunohistochemical analysis (arrows). Cell nuclei are stained with DAPI (blue). G-I: Western blot analysis of NMII isoforms in E11.5 *Myh10*∆ heterozygous control and homozygous mutant whole heart protein extracts. Protein extract from adult mouse lung was run as a positive (+ve) control. NMIIA and IIB are found in relative abundance at this developmental stage in heterozygous hearts (G and H). As expected, NMIIB is absent from mutant samples (H, lane 3), which also show a reduction in NMIIA (G, lane 3). NMIIC is not detectable in either control or mutant samples (I). Protein extracts pooled from at least n = 3 hearts for each genotype. J-O: Immunocytochemistry for the predominant IIA and IIB isoforms in *Myh10*∆ heterozygote (J, K, M and N) and mutant (L, O) epicardial cell cultures from E11.5 heart explants. Note localisation of NMIIA to the periphery of epicardial cells (J, K, arrows), whilst NMIIB is localised throughout the whole cell body (M, N, asterisks), and is frequently associated with actin stress fibres (M, N, arrow). Consistent with western blot analysis (H), NMHC IIB is not detectable in mutant samples (O). Images representative of cell cultures obtained from four hearts per genotype. P: NMIIB immunofluorescence (green) in E11.5 control sample. NMIIB is expressed in the epicardium (arrow). Nuclei are labeled with DAPI (blue). Q: NMIIB expression in *Myh10*∆ homozygous mutant heart at E11.5. R: NMIIB immunofluorescence (green) at E14.5 in control sample. NMIIB is highly expressed in the epicardium (arrow). Nuclei are labeled with DAPI (blue). S: NMIIB expression in *Myh10*∆ homozygous mutant heart at E14.5. NMIIB is not detectable in *Myh10*∆ homozygous mutant samples, consistent with western blot (H) and cell culture results (O). Scale bars: A-F, J, L, M and O = 100 μm, K, N, P-S = 50 μm. Images in A-C and D-F obtained at same magnification. Abbreviations: ∆: *Myh10*∆.

We sought to establish why the epicardium in particular demonstrates abnormalities following NMHC IIB ablation that are not exhibited in other tissue types. There are three NMHC II isoforms, IIA, IIB, and IIC, encoded by *Myh9*, *Myh10* and *Myh14* respectively in the mouse [20, 21]. The NMHC IIB null mouse displays developmental defects primarily in the brain and heart, which the authors attribute to an enrichment of NMHC IIB in these tissues [33, 37, 54]. However, the relative expression levels of individual NMHC II isoforms have not been explored specifically within the embryonic epicardium. We analysed NMHC II protein levels in control embryonic hearts using immunofluorescent microscopy. This analysis revealed that NMHC IIB is the predominant NMHC II isoform found in the E14.5 heart, and moreover, within the epicardium (Fig 8A–8C, arrows). All NMHC II isoforms were detectable in the developing lung (Fig 8D–8F, arrows), as previously described [37]. In addition, at E11.5 we found NMHC IIB together with IIA, were abundant in whole heart protein extracts probed by western blotting (Fig 8G and 8H). Somewhat surprisingly, we found that levels of NMHC IIA appeared to be diminished in NMHC IIB ablated samples (Fig 8G), whilst NMHC IIC was not detectable in either control or mutant preparations (Fig 8I). In light of this, we sought to determine the relative abundance and subcellular localisation of NMHC IIA and IIB in enriched epicardial cell cultures derived from E11.5 heart explants by immunocytochemistry. In both control and mutant cultures, NMHC IIA appeared to be primarily localised to the cell periphery in cells at both the leading edge and within the culture monolayer (Fig 8J–8L, arrows). In comparison, not only was fluorescent signal notably increased for NMHC IIB (Fig 8M and 8N), its subcellular localisation appeared to be more diffuse throughout the cell body (Fig 8N, asterisks), and frequently associated with cytoskeletal stress fibres (Fig 8N, arrows). As expected, NMHC IIB was not detectable in NMIIB ablated cultures (Fig 8O). Interestingly, mutant epicardial cells did not display either an increase in NMHC IIA signal intensity, or altered NMHC IIA subcellular distribution when compared to controls (Fig 8L compared to 8J). Higher magnification images of control hearts at E11.5 and E14.5 demonstrate NMHC IIB protein localisation to the epicardium (Fig 8P and 8R), with higher levels of epicardial expression found at E14.5 (Fig 8R) as compared to E11.5 (Fig 8P). We did not detect any immunofluorescent signal in *Myh10∆* homozygous mutant hearts at either E11.5 or E14.5 using the NMHC IIB C-terminal antibody (Fig 8Q and 8S). Together, these data suggest that NMHC IIB is expressed at both a higher abundance in the epicardium, and in distinct subcellular regions to other NMHC II isoforms. Consequently, NMIIB may be serving a specialised function in the epicardial cells that cannot be compensated by other NMII isoforms when NMIIB is lost, thus exposing epicardial dysfunction.

### *EHC* epicardial cells do not exhibit migration defects *in vitro*

**Fig 9:**
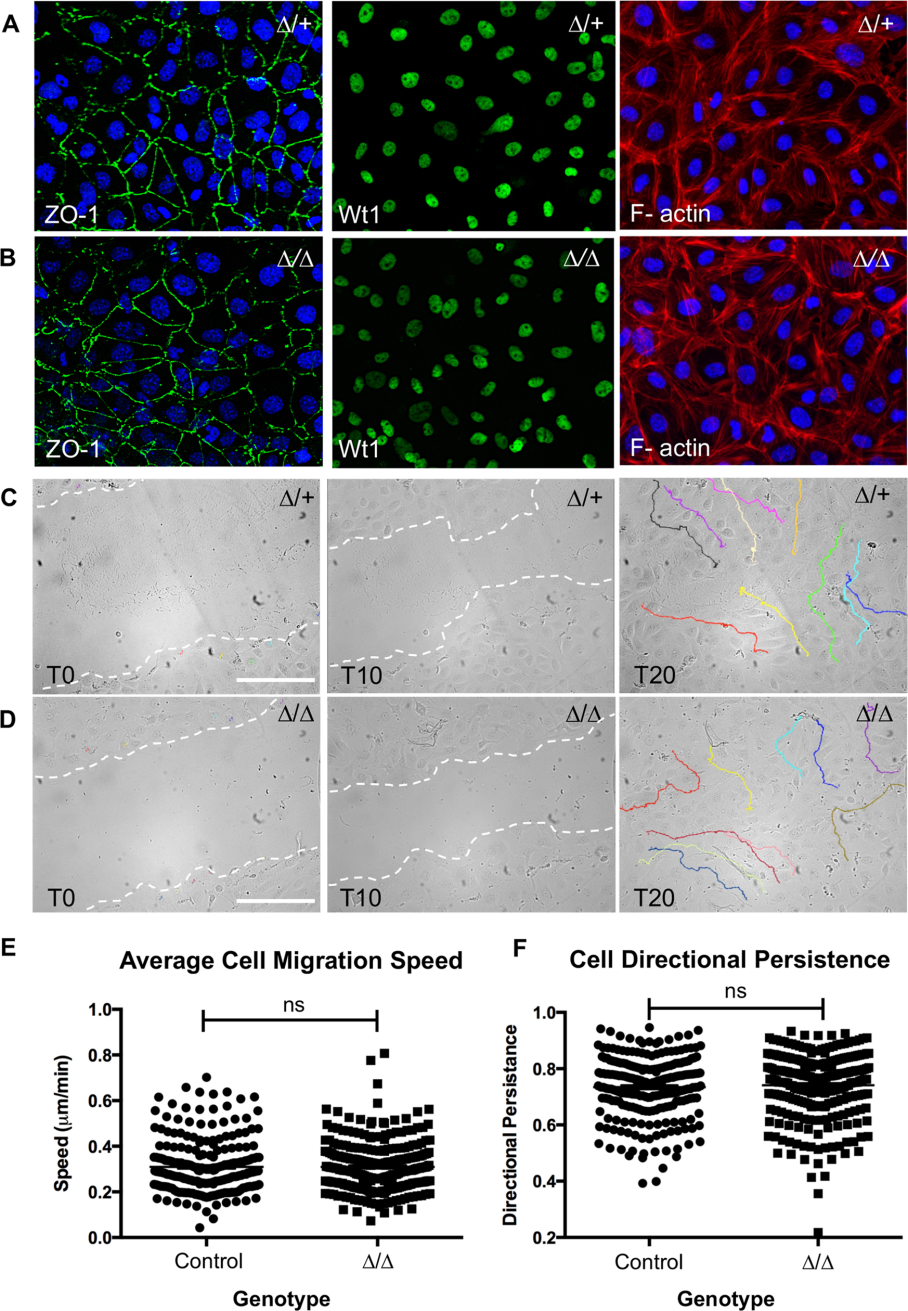
NMHC IIB ablated epicardial cells do not show migration defects *in vitro*. A: Cells cultured from E11.5 *Myh10*∆/+ heterozygote and B: *Myh10*∆ homozygous mutant heart explants form an epicardial monolayer. Epicardial status was confirmed by positive staining for the epithelial marker ZO-1 (left panels), and the epicardial marker, Wt1 (centre panels). Rhodamine-phalloidin staining of the actin cytoskeleton also revealed characteristic ‘cobblestone’ morphology, indicative of an epithelial cell population (right panels). C: Control and D: mutant epicardial monolayers were scratched at T0 (left panels) and imaged at 10-minute intervals for 20 hours. The migration of ten cells per field of view was tracked using ImageJ (right panels, coloured lines). E: Graph showing the subsequent comparison of mean cell migration speed (control = 0.3099μm/min, mutant = 0.3098μm/min, Mann-Whitney U-test p = 0.6717). F: Graph showing the comparison of cell migration directional persistence (control = 0.7342, mutant = 0.7411, Mann-Whitney U-test p = 0.2494). Total tracked cells = 240 control and 270 mutant. Cultures were generated from at least four hearts for each genotype. Control refers to data compiled from both wild type and ∆/+ genotypes. Scale bars: 250 μm. Abbreviations: ∆: *Myh10*∆.

The formation of the epicardium is wholly reliant upon the migration of cells from the PEO to the surface of the developing myocardium [8]. NMHC IIB has been shown to play an important role in cell migration, through the generation of traction forces, and guidance of directional persistence [20, 21, 28, 36]. In light of this, we sought to investigate whether NMHC IIB null epicardial cells demonstrated motility defects *in vitro* by performing a scratch wound assay on epicardial cells cultured from embryonic heart explants as previously described [55]. Epicardial cells from control (+/+ or *Myh10*∆/+) and NMHC IIB null (*Myh10*∆/*Myh10*∆) E11.5 hearts were enriched and cultured for 48 hours to confluence on gelatin-coated 24-well plates. The *Myh10*∆ line was used for these experiments to expedite genotyping. The epicardial nature of the resultant cell populations was confirmed by immunostaining for the epithelial marker, ZO1, epicardial marker, Wt1 (Wilms Tumour 1), and the epithelial ‘cobble-stone’ morphology of filamentous actin staining with rhodamine phalloidin (Fig 9A and 9B). These epicardial monolayers were scratched with a P10 pipette tip and wound closure was imaged over a 20-hour period (S1 and S2 Movies). For each image series, we identified 10 cells at the leading edge of the wound at T0, and manually tracked their migration (Fig 9C and 9D). Cell migration speed and directional persistence were subsequently analysed using ImageJ. Somewhat surprisingly, we found that NMHC IIB null epicardial cells exhibited normal migratory behaviour when compared to controls, with no significant difference in either the average migration speed (Fig 9E, Mann-Whitney, p = 0.6717), or directional persistence (Fig 9F, Mann-Whitney, p = 0.2494). This finding suggests that epicardial cells do not require NMHC IIB to exhibit normal migratory behaviour *in vitro*.

### *EHC* epicardial-derived cells demonstrate disrupted functionality *in vivo*

**Fig 10:**
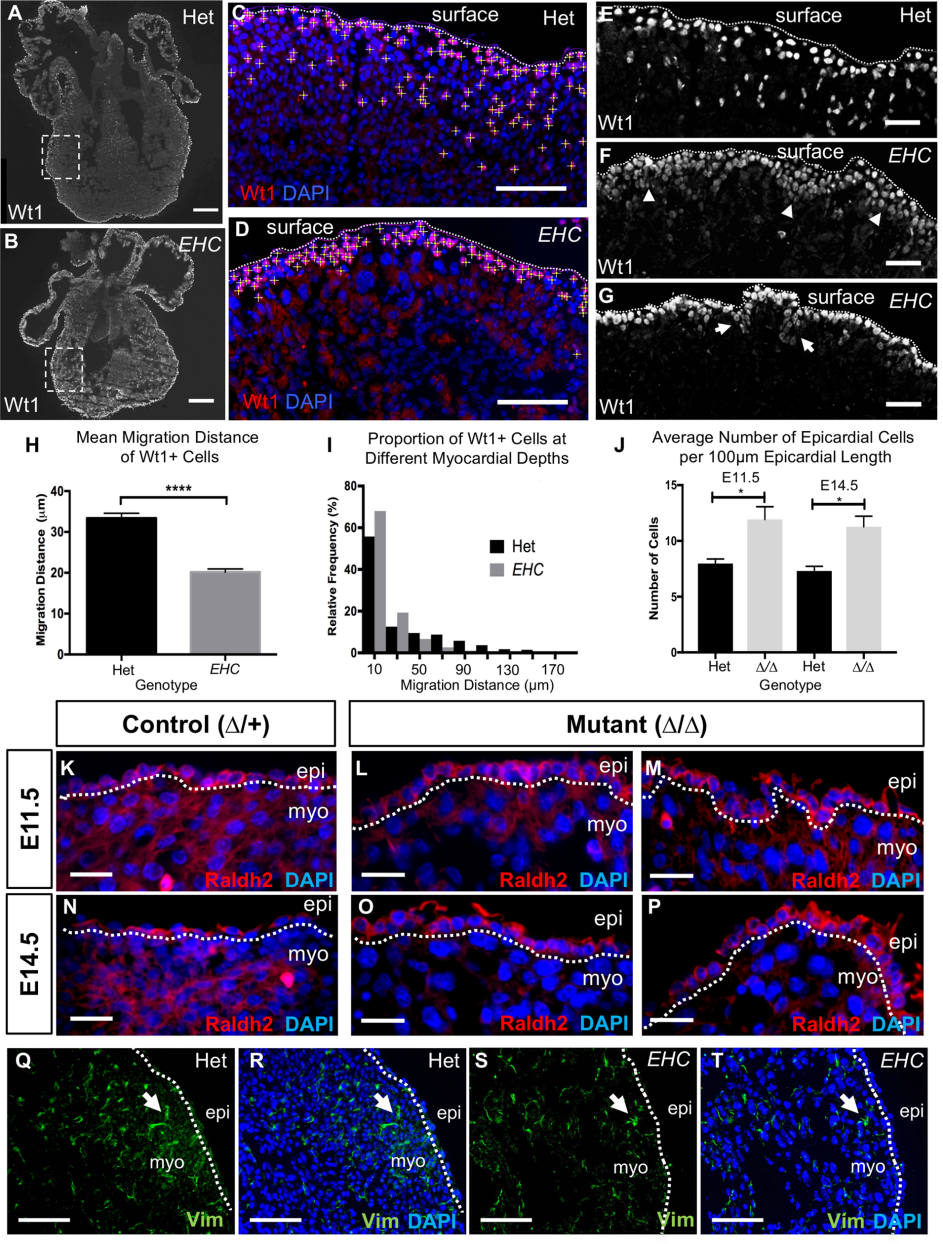
*EHC* epicardial-derived cells show defective migration *in vivo*. A: Representative images of Wt1 immuno-stained coronal cryo-sections of *EHC* heterozygous and B: homozygous mutant E14.5 hearts. The dashed box indicates the area of ventricular wall used for measurement analysis in (C) and (D). C: Wt1 positive epicardial-derived cells were marked with yellow crosshairs in heterozygous and D: mutant hearts. The distance between these cells and the cardiac surface (C-G: dashed line) was manually measured using ImageJ. E: Wt1 positive cells (white) in the heterozygous heart can be seen at the cardiac surface (dashed line) indicating localization in the epicardium, as well as in deeper cardiac tissue below the dashed line. F: *EHC* mutants have Wt1 positive cells (white) primarily at the cardiac surface (dashed line), but these cells are organised into abnormal clusters (arrowheads), with few cells at deeper positions in the underlying myocardium. G: Wt1 positive cells surround the ventricular surface blisters (arrows) present in *EHC* mutant hearts. H: Graph comparing the mean migration distance of Wt1 positive cells: Het = 33.37 μm (+/-1.197 μm SEM, n = 975), mutant = 20.14 μM (+/-0.8136 μm SEM, n = 1180), Mann-Whitney U-test p = <0.0001). I: Histogram showing the relative frequency of Wt1 positive cells at increasing depths within the myocardial wall. A higher proportion of mutant Wt1 positive cells reside in the subepicardial region (<50 μm from the apical epicardial boundary) compared to controls. J. Comparison of the mean number of Raldh2 expressing cells found on the cardiac surface of hearts per unit of epicardial length for each genotype at E11.5 and E14.5. Mean number of epicardial cells/100μm in E11.5 controls = 7.975 (+/- 0.4008, n = 4) and mutants = 11.93 (+/- 1.145, n = 7); Mean number of epicardial cells/100μm in E14.5 controls = 7.3 (+/- 0.4243, n = 4) and mutants = 11.27 (+/- 0.9502, n = 10). A significant difference is detected between genotypes at E11.5 (2-tailed unpaired t test, p = 0.0335) and E14.5 (2-tailed unpaired t test, p = 0.0257). Error bars represent standard error of the mean. K: Raldh2 protein localisation (red) in *Myh10∆* heterozygous control and L-M: *Myh10∆* homozygous mutant heart at E11.5. Sections from two different embryos are shown. N: Raldh2 protein localisation (red) in *Myh10∆* control and O-P: *Myh10∆* homozygous mutant heart at E14.5. Sections from one embryo at different cardiac depths are shown (O-P). Q: Vimentin immunofluorescence in E14.5 control heart. Spindle-shaped mesenchymal cells are present (arrow). R: Merged image showing nuclei (blue). S: Vimentin immunofluorescence in E14.5 *EHC* mutant heart. Spindle-shaped mesenchymal cells are present (arrow). T: Merged image showing nuclei (blue). The epicardial boundary with the myocardium is labeled with a dashed line. Scale bars: A and B = 250 μm, C, D, Q-T = 100 μm, E-G = 50 μm, K and N = 25 μm. Abbreviations: epi: epicardium, myo: myocardium.

It is well documented that during development, a sub-population of epicardial cells undergo EMT and give rise to epicardial-derived cells that have acquired the ability to invade the underlying myocardium, where they differentiate into multiple cell lineages, including interstitial and perivascular fibroblasts and vascular smooth muscle cells [15, 45]. In addition to this cellular contribution, it is suspected that EPDCs regulate myocardial development through expression of paracrine signalling molecules [56–60]. It is clear that the correct execution of epicardial EMT is essential for both coronary and myocardial development. Since we did not detect epicardial motility defects *in vitro*, we examined epicardial cell migration into the underlying cardiac tissues *in vivo* by evaluating the localisation of cells expressing the epicardial marker Wt1 in *EHC* mutant and control embryos (Fig 10A–10G). Measurements of the distance between Wt1 positive cells in the ventricular myocardium of E14.5 hearts (Fig 10C and 10D, yellow crosshairs) and the cardiac surface indicated that *EHC* EPDCs had not penetrated as deeply into the myocardium as EPDCs in control hearts (Fig 10H, Mann Whitney, p<0.0001). Consequently, the majority of *EHC* EPDCs resided in a tightly restricted region of the subepicardial space (Fig 10F and 10I) or specifically around the blood-filled ventricular vesicles apparent in mutant hearts (Fig 10G). To confirm that the cells we detected at the cardiac surface were epicardial cells, we used the marker Raldh2. We found that Raldh2 expressing cells are located at the cardiac surface at E11.5 and E14.5 in both heterozygous control and *Myh10∆* homozygous mutants (Fig 10K–10P). Quantification of the number of Raldh2 positive cells per length of epicardium reveals a significant increase in epicardial cell number in *Myh10∆* homozygous mutants at both E11.5 and E14.5 when compared to control littermates (Fig 10J). We also evaluated vimentin expression at the cardiac surface in *EHC* mutant embryos and heterozygote controls (Fig 10Q–10T). We did not detect increased vimentin staining in *EHC* mutant embryos, indicating that there is not an increase in mesenchymal cell populations at the surface of the *EHC* heart. These *in vivo* results mirror the defects exhibited in other mouse models with compromised epicardial cell function [32, 61–63]. In addition, an *in vivo* cell migration defect may well be predicted from previous studies in which NMHC IIB is ablated, or its activation inhibited [28, 32]. Together, these data provide a strong evidence base to support our hypothesis that epicardial cell abnormalities contribute to the coronary vessel defects displayed by *EHC* mutant mice.

### The ECM environment is altered in NMHC IIB mutant hearts

**Fig 11:**
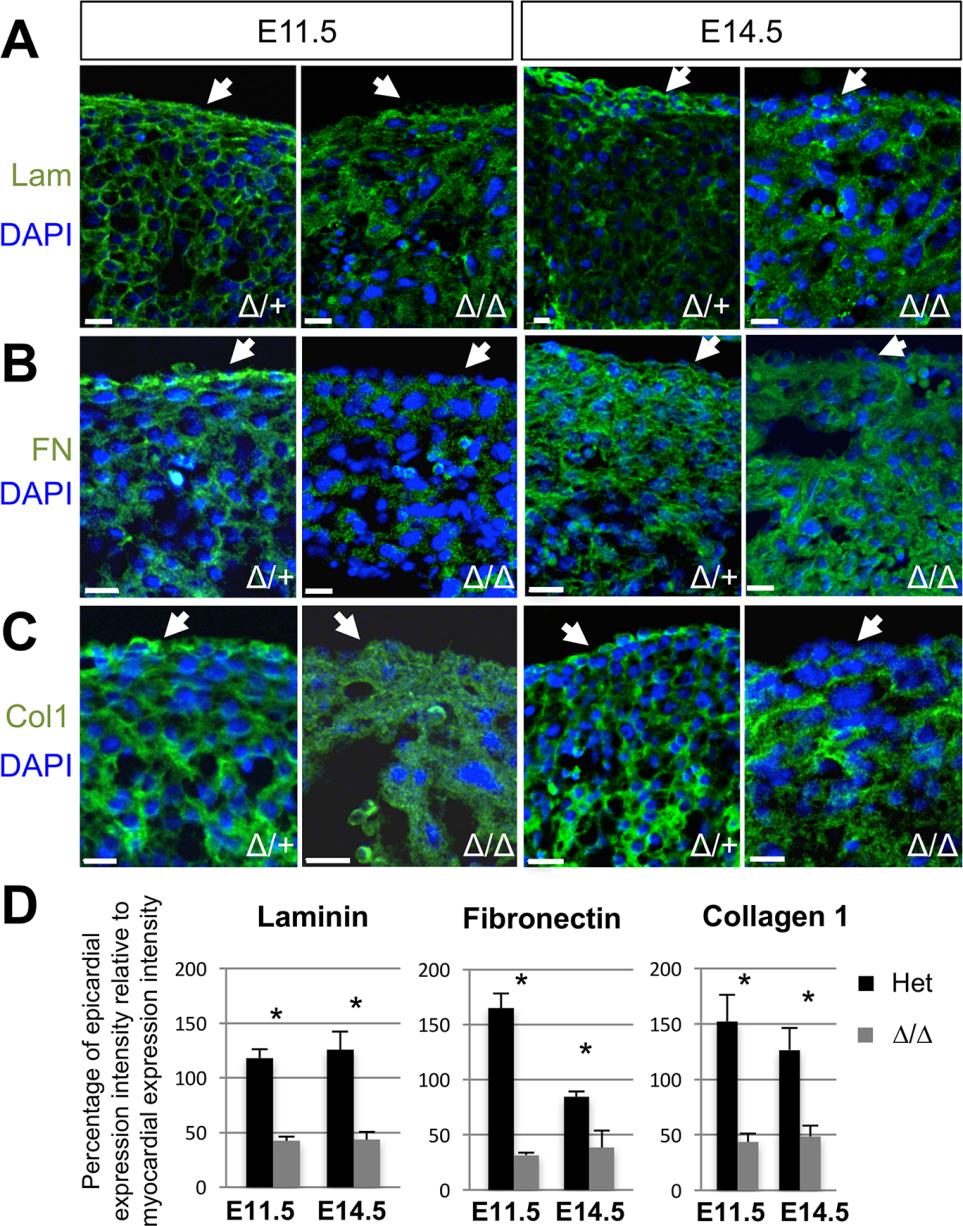
Examination of ECM composition in *Myh10*∆ control and mutant embryos. A: Laminin immunofluorescence (green) is present at E11.5 and E14.5 in the *Myh10∆* heterozygous ventricular myocardium and is continuously distributed in the epicardium (arrow). *Myh10∆* homozygous mutants show reduced laminin immunoreactivity in the epicardium (arrow) at both E11.5 and E14.5. Nuclei are marked with DAPI (blue). B: Fibronectin immunofluorescence (green) is present at E11.5 and E14.5 in the *Myh10∆* heterozygous ventricular myocardium and is continuously distributed in the epicardium (arrow). *Myh10∆* homozygous mutants appear to have reduced signal, especially in the epicardium (arrow) at E11.5 and E14.5. Nuclei are marked with DAPI (blue). C: Collagen 1 immunofluorescence (green) is present at E11.5 and E14.5 in the *Myh10∆* heterozygous ventricular myocardium and is continuously distributed throughout the epicardium (arrow). *Myh10∆* homozygous mutants have reduced collagen 1 signal in the epicardium (arrow) at E11.5 and E14.5. Nuclei are marked with DAPI (blue). Genotypes and developmental stages are labeled on the image. D: Quantification of the ratios of expression levels of epicardial immunofluorescence intensity to myocardial immunofluorescence intensity for each protein. Three embryos of each genotype were analysed, with measurements taken from five different areas of three different sections from each embryo. All markers and time points showed a statistically significant reduction (*) in immunofluorescence intensity of the epicardium relative to the myocardium in *Myh10∆* homozygous mutants as compared to controls (two-tailed t-test, p<0.05). Ratios of expression for each genotype were compared to each other for each marker and developmental stage. Error bars represent standard deviation. Genotypes, markers and stages are labeled on the graphs. Scale bars: 15 μm. Abbreviations: Lam: laminin, FN: fibronectin, Col1: Collagen I.

Following the observation that *Myh10∆* homozygous mutant epicardial cells display altered migration *in vivo*, but not in scratch wound assays *in vitro*, we hypothesised that the coronary vessel development defects in the *EHC* and *Myh10∆* mutant embryos might be due to an altered *in vivo* environment affecting epicardial derived cell migration. We have shown that Alcian blue staining for GAGs was reduced in the *EHC* subepicardial ECM (Fig 7G and 7J). Since GAGs constitute a major molecular component of the extracellular matrix, we hypothesised that the loss of NMIIB disrupts ECM protein distribution in the developing heart, thus hindering epicardial cell motility or migration *in vivo*. We therefore evaluated the expression of laminin and fibronectin, ECM components documented to be expressed in the human embryonic heart [64]. Fibronectin is of interest particularly in the developing epicardium, since it has been shown to be required for directional persistence during epicardial cell migration [65]. In the mouse heart, fibronectin has been reported to localise to the epicardium from E12.5 to E16.5, where it co-localises with collagen I [66]. To evaluate ECM distribution we performed immunofluorescence for laminin, fibronectin, and collagen I in *Myh10∆/+* control and *Myh10∆* homozygous mutant hearts at E11.5 and E14.5. We found that laminin, fibronectin and collagen I were abundantly present in the subepicardial ECM of control hearts, and indeed, throughout the ventricular myocardium (Fig 11A–11C, arrows). The distribution of these ECM components was altered in *Myh10∆* homozygous mutant hearts, with little detectable staining in the epicardial layer and generally reduced levels throughout the heart (Fig 11A–11C, arrows). Quantification of the staining intensity in the epicardial region relative to staining intensity in the myocardial region on the same tissue section was performed. All ECM components analysed demonstrated a significantly reduced ratio of expression in *Myh10∆* homozygous mutants at E11.5 and E14.5 (Fig 11D), suggesting that the extracellular environment of the mutant heart has been altered. Furthermore, *Myh10∆* homozygous mutant epicardial explants cultured independently from myocardial cells display an altered pattern of fibronectin distribution, with poor organisation and a reduced network of fibrils (S6 Fig), confirming that mutant epicardial cells have an impaired ability to produce fibronectin.

### Analysis of epicardial cell apoptosis, proliferation, and signaling in NMHC IIB mutants

**Fig 12:**
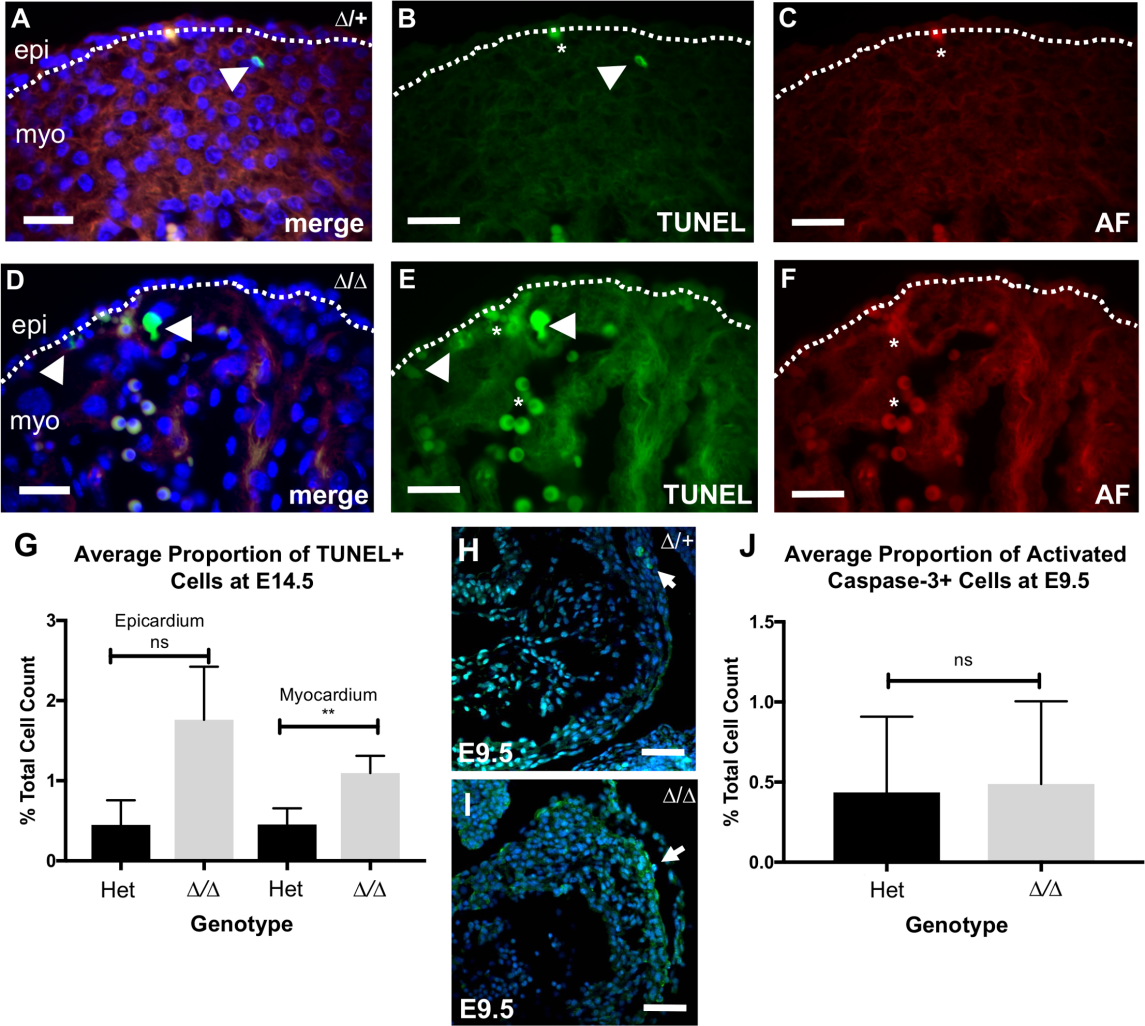
Apoptosis in *Myh10∆* mutant hearts. A: TUNEL analysis (green) of *Myh10∆* heterozygous heart at E14.5 shown with nuclei stained with DAPI (blue). B: Single channel of TUNEL staining from (A). Apoptotic cells (arrowhead) are present along with autofluorescent erythrocytes (asterisk). C: Autofluorescence of erythrocytes (red) from (A). D: TUNEL analysis (green) of *Myh10∆* homozygous mutant heart at E14.5 shown with nuclei stained for DAPI (blue). E: Single channel of TUNEL staining from (D). Apoptotic cells (arrowhead) are present along with clusters of erythrocytes (asterisks). F: Autofluorescence of erythrocytes (red) from (D). G: Graph comparing mean number of TUNEL positive cells as a proportion of total cells in either the epicardium, or myocardial wall. Mean percentage of apoptosis in control = 0.4484% (+/- 0.3082%), and mutant epicardium = 1.762% (+/- 0.662%) (unpaired 2-tailed Mann-Whitney U-test, p = 0.0744). Mean percentage of apoptosis in control = 0.4532% (+/- 0.204%), and mutant myocardial wall = 1.097% (+/- 0.2132%) (unpaired 2-tailed Mann-Whitney U-test, p = 0.0053). Three hearts per genotype and six non-consecutive sections per heart were analysed. H: Sagittal section of control and I: *Myh10∆* homozygous mutant hearts at E9.5 showing immunofluorescence for activated Caspase-3 (turquoise, arrows), with nuclei visualised with DAPI (blue). J: Graph comparing mean number of activated caspase-3 positive cells as a percentage of total cells in cardiac sections of control and mutant E9.5 hearts. Mean percentage of apoptosis in control = 0.435% (+/- 0.0.1579%), and mutant hearts = 0.4885% (+/- 0.1722%). Three hearts were examined per genotype, and three sections were analysed for each heart. There is no significant difference in apoptosis between the two genotypes (unpaired 2-tailed Mann-Whitney U-test, p = 0.9292). Error bars in G and J represent standard error of the mean. Scale bars: A-F = 25 μm, H-I = 50 μm.

Correct deposition of the cardiac ECM is necessary for appropriate cardiac development [67]. In the adult heart, alterations in ECM composition are associated with apoptosis [68]. After detecting ECM defects in NMHC IIB ablated mutants, we therefore assessed apoptosis in the *Myh10∆* homozygous mutant heart at E14.5 using a TUNEL assay, and compared our findings to control samples (Fig 12A–12G). At this developmental stage, control hearts displayed low levels of apoptosis in both the epicardium and underlying myocardium (Fig 12A and 12B). We found a small but statistically significant increase in the average number of apoptotic cells within the mutant myocardium, compared to control littermates (Fig 12G; Mann Whitney U test, p<0.006). In contrast, whilst the mutant epicardium also displayed elevated apoptosis rates when compared to controls, this finding was not statistically significant (Fig 12G; Mann Whitney U test p = 0.07). Similarly, no statistically significant difference was observed between the number of apoptotic cells in activated caspase-3 stained control (Fig 12H) and mutant (Fig 12I) hearts during early development at E9.5 (Fig 12J; unpaired 2-tailed Mann-Whitney U-test, p = 0.9292). These data suggest that loss of NMHC IIB does not cause significant epicardial apoptosis.

**Fig 13:**
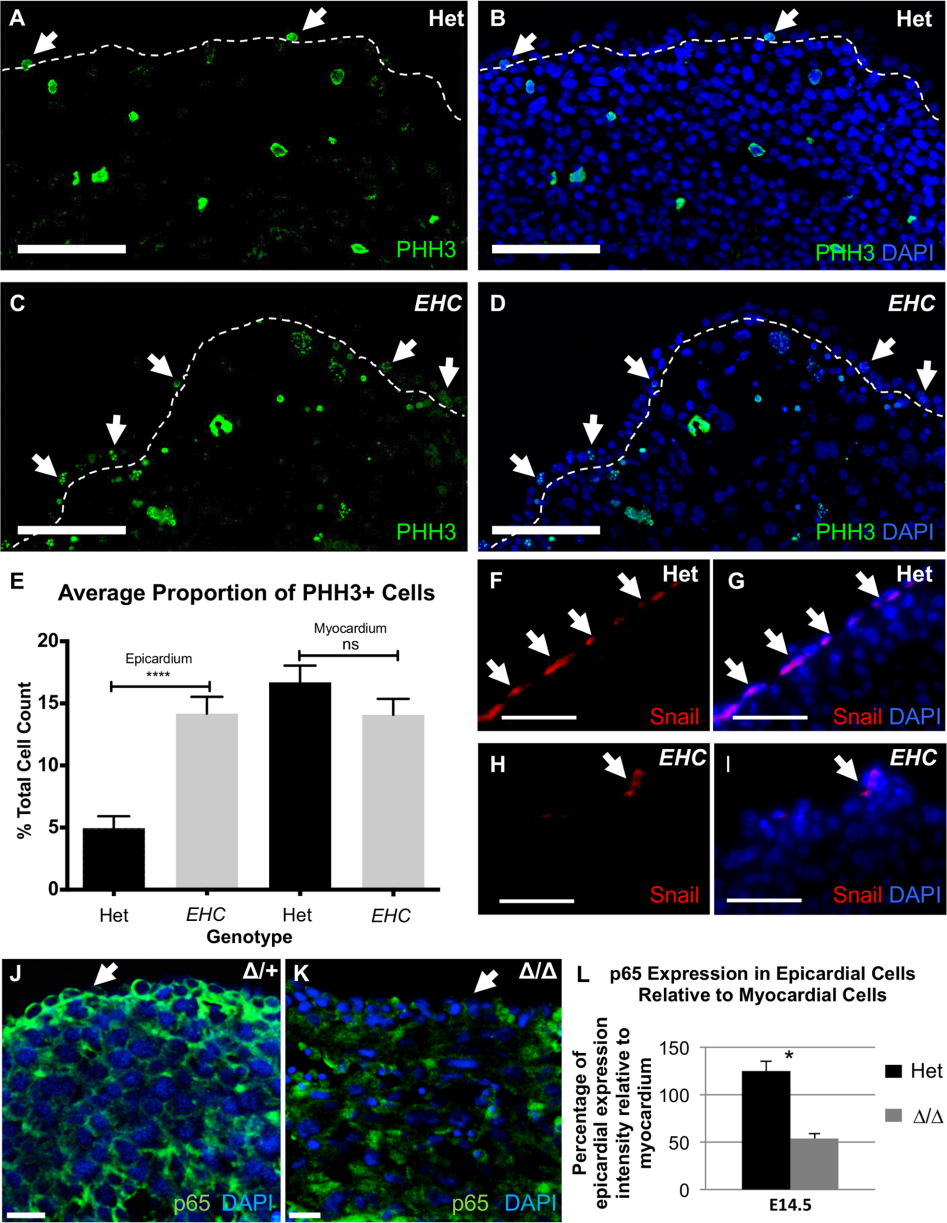
*EHC* epicardial cells show defects in EMT. A-B: *EHC* Heterozygous and C-D: *EHC* homozygous mutant E14.5 heart cryo-sections were stained for the proliferation marker, PHH3 (green). Proliferating epicardial cells are indicated by arrowheads. The dashed line indicates the boundary between the epicardium and myocardium. Cell nuclei were stained with DAPI (blue). E: Graph comparing the proportions of proliferating cells (as a percentage of total cells) in both the epicardium and myocardium between controls and homozygous mutants. Average proportion of proliferating cells in control = 4.953% (+/- 0.9674%) and *EHC* mutant epicardium = 14.17% (+/- 1.356%) (unpaired 2-tailed t-test, p = <0.0001). Average proportion of proliferating cells in control = 16.70% (+/- 1.348%), and mutant myocardium = 14.09% (+/- 1.275%) (unpaired 2-tailed t-test, p = 0.1684). Error bars represent standard error of the mean. F-I: Localisation of the EMT marker, Snail in the control and mutant epicardium (arrowheads). Cell nuclei are indicated by DAPI staining (blue). The *EHC* epicardium shows a reduction in the number of cells in which EMT is activated. J: p65 immunofluorescence (green) in *Myh10∆* heterozygous heart showing expression in the epicardium (arrow). Nuclei are labeled with DAPI (blue). K: p65 immunofluorescence (green) in *Myh10∆* homozygous mutant heart showing diminished immunoreactivity in the epicardium (arrow). L: Quantification of p65 immunofluorescence intensity in the epicardium relative to the myocardium. Genotypes are labeled on the graph. A statistically significant reduction in p65 epicardial expression is seen in *Myh10∆* homozygous mutants (t-test, p = 0.003). Error bars represent standard deviation. Images were analysed following the same methodology as reported for Fig 11D. Scale bars: A-D = 100μm, F-I = 50μm, J-K = 15 μm. Abbreviations: Wt1: Wilms tumour 1, PHH3: phosphohistone H3.

We next sought to establish whether the function of *EHC* epicardial cells was compromised prior to EPDC migration, specifically focusing on the process of epicardial EMT. Analysis of the proliferation marker phosphohistone H3 (PHH3) revealed a distinct increase in proliferation within the epicardium of *EHC* mutants at E14.5 as compared to controls (Fig 13A–13D, arrows; Fig 13E, unpaired t-test, p<0.0001). We found no significant difference in cell proliferation in the underlying cardiac tissue between controls and *EHC* mutant hearts (Fig 13E, unpaired t-test, p = 0.1684), nor in total cardiac tissue between control and *Myh10∆* homozygous mutant hearts at E9.5 (S6C Fig, unpaired t-test, p = 0.57). This finding is consistent with a reduced incidence of EMT induction, as cells undergoing EMT attenuate cell division in favour of changes to cell morphology [69]. Correspondingly, we examined the localisation of the EMT marker, Snail, within the epicardium at E14.5. Similarly, we found that the *EHC* mutants demonstrated a reduction in the number of Snail positive epicardial cells (Fig 13F–13I, arrowheads). Recently it has been reported that NF-κB signaling is required downstream of TGFβ and PDGF inputs to mediate epicardial cellular changes associated with EMT [70]. The NF-κB component p65 is expressed in the mouse epicardium during EMT [70]. We examined p65 expression at E14.5 in heterozygote control and *Myh10∆* homozygous mutant hearts (Fig 13J–13K) and calculated the ratio of epicardial staining intensity compared to myocardial staining intensity. We found a statistically significant reduction in the ratio in *Myh10∆* mutant embryos, indicating reduced p65 expression in the mutant epicardium (Fig 13L). Together, this evidence is highly suggestive of epicardial EMT dysregulation specifically in the mutant epicardium. This result suggests an important role for NMHC IIB in the promotion or execution of EMT, via NF-κB signaling, that to our knowledge has not been reported previously.

## Discussion

The present study demonstrates a requirement for NMHC IIB during the formation of the mammalian coronary vasculature. Here, we report the characterisation of a mutant mouse line, generated from a balancer chromosome *ENU* mutagenesis screen [19], that displays embryonic hydrocephalus and cardiac defects. The *EHC* point mutation in *Myh10* results in a global loss of NMIIB function, as confirmed by genetic complementation of the *EHC* allele *in trans* to the *Myh10∆* allele. Notably, the *Myh10* gene is located approximately 1 Mb outside the *Trp53* endpoint of the balancer chromosome interval [19]. In breeding the *EHC* mutants we have only had 1 animal out of more than 1180 in total that showed recombination between the *Myh10* mutation and the balancer chromosome end point, indicating that the balancer chromosome can be used to maintain balanced heterozygous stocks for embryonic lethal mutations in genes located outside the balancer interval.

Our studies of the *EHC* mouse have made advances in understanding the essential role undertaken by NMHC IIB during cardiogenesis. The *EHC* mutant coronary vessel abnormalities are markedly similar to phenotypic observations of mice in which the epicardium has been specifically disrupted [29, 31, 63], and are consistent with a primary defect in epicardial cell function. We have shown that *EHC* mutant epicardial cells have a highly perturbed morphology, impaired epicardial EMT, and disrupted subepicardial ECM composition, in addition to reduced migration of EPDCs into the myocardium. The absence of mature coronary vessels in *EHC* mutants suggests that *in vivo* these dysfunctional EPDCs are incapable of contributing to the vascular network. A complementary study has demonstrated that coronary vessel development is impaired when *Myh10* is deleted specifically in the epicardium [53], consistent with our conclusion that epicardial defects underpin disrupted coronary vessel development in *EHC* mutant mice.

NMHC IIB plays a key role in a broad variety of fundamental cellular processes [20, 21]. Similarly, the defects we have documented encompass a range of physiological events, including, but not limited to: cell migration, adhesion, proliferation and apoptosis. Our finding that *EHC* mutants display defects in epicardial EMT is of particular interest, as other fundamental developmental processes that depend upon the correct initiation and execution of EMT (e.g. gastrulation, craniofacial development) occur in the *EHC* embryo. Indeed, formation of the atrioventricular valves, a process dependent upon endocardial cushion EMT [13], is evident in *EHC* mutant hearts (S5 Fig). We have demonstrated that NMHC IIB is the predominant NMHC II isoform in epicardial cells both *in vivo* and *in vitro*, and that NMHC IIB is localised to distinct subcellular regions (in agreement with the findings of Lo et al., [28], and Ma et al., [53]). Therefore, it may be hypothesised that NMHC IIB plays critical roles in epicardial and cardiac development, which cannot be replaced by other NMHC II isoforms when NMHC IIB function is globally ablated. The observation that loss of NMHC IIB leads to decreased expression of NMIIA presents the possibility of altered transcriptional regulation in *EHC* mutants, which needs to be further explored to understand the roles of NMIIB in epicardial cell function, and embryonic development as a whole. Notably, unlike NMHC IIA or IIC, NMHC IIB displays cell type and cell-cycle specific mechanosenstivity [71], which may be specifically altered in mutant epicardial cells during development.

The myocardium also plays a role in epicardial cell behaviour, through the secretion of paracrine signalling molecules which traverse the subepicardial ECM and communicate with the epicardium to orchestrate correct epicardial function [13, 15, 50, 60]. The requirement for NMHC IIB in myocardial development is well documented, with previous reports observing myocardial disorganisation, cardiomyocyte cytokinesis defects, and a reduction of the myocyte population in NMHC IIB null hearts [23, 33, 36, 37]. However, cardiomyocyte-specific *Myh10* ablated mice are viable, and importantly, demonstrate a reduced instance of VSD and the complete absence of DORV [35]. Here we report that NMHC IIB is not required within the cardiomyocyte population for coronary vessel development. This finding suggests that the severe morphological defects present in NMHC IIB null hearts are caused by loss of NMHC IIB from other cardiac cell populations. It has recently been demonstrated that epicardial-specific deletion of *Myh10* does impair coronary vessel development [53], although the reported defects are not as severe as we have documented here for the *EHC* mutant, *EHC/Myh10∆* compound heterozygote, or *Myh10∆* homozygous mutant embryos. Further investigation is required to determine if these phenotypic differences are due to differences in assays used or due to requirements for NMHC IIB in multiple cell types during coronary vessel development.

NMHC IIB has been directly shown to be important in cell migration as a key component of the actin-myosin cytoskeletal machinery [20, 21, 28, 36]. Our use of the epicardial cell culture model shows that the migration of *EHC* epicardial cells progresses unimpeded *in vitro*, both in the context of epicardial cell outgrowth from embryonic heart explants, and in a wound-healing assay. Moreover, cultured primary epicardial cells have been previously shown to express *Snail* [72], indicating that the process of epicardial cell migration from explant heart tissue *in vitro* involves EMT activation. A key difference between the explant model and the *EHC* mutant is the context of the extracellular environment. We performed our explant assays on gelatin-coated plates, and it has been demonstrated that the provision of exogenous ECM substrate can compensate for migration defects in cells with ECM production deficiencies [73]. It is known that the subepicardial ECM plays an important role in epicardial function, in both the adhesion of the epicardial monolayer to the myocardium and in facilitating molecular communication through the subepicardial space [13, 50, 60, 74]. During EMT, TGFβ signaling (a key input for epicardial EMT [60]) is known to be affected by ECM substrate rigidity [75]. Additionally, alterations in cell tension, provoked by changes in the ECM, can disrupt nuclear architecture and chromatin structure, with subsequent effects on transcriptional regulation [76]. Disruption of the subepicardial ECM as seen in *EHC* and *Myh10∆* mutants may therefore alter the ability of *EHC* EPDCs to migrate into the myocardium.

The detection of abnormalities in EMT signaling, indicated by increased epicardial cell proliferation and reduced Snail expression in *EHC* mutants, is surprising, as NMHC IIB would be expected to be a downstream effector of cell motility in EMT. Although EMT defects have been detected in the epicardial-specific *Myh10* knock out [53], comparison of the results of that study with ours is complicated by the use of different assay methods and the potential for requirements for NMHC IIB in non-epicardial cells to influence our findings. However, our results suggest that the loss of NMHC IIB disrupts processes required for EMT signaling, through the NF-κB pathway acting downstream of TGFβ and PDGF inputs [70]. PDGF is produced in the myocardium and serves as a paracrine signal to promote epicardial EMT [60]. Phenotypically, the epicardial cell morphology defects we note from EM studies are similar to those reported for a PDGFRβ knock out [63]. Interestingly, it has previously been shown that changes in ECM composition, particularly collagen, can alter PDGF responsive gene activation during wound healing [77]. We have detected reduced p65 in *Myh10∆* mutants, indicative of impaired NF-κB pathway activation. Since NF-κB pathway activation occurs in response to PDGFBB ligand inputs [70], we speculate that the observed alterations in the subepicardial ECM of mutant embryos may impede PDGF signalling, which subsequently hinders NF-κB pathway activation, thus contributing to epicardial EMT dysregulation in *EHC* and *Myh10∆* homozygous mutant embryos.

In summary we have demonstrated a requirement for NMHC IIB to generate the appropriate cardiac extracellular matrix environment at the subepicardial space. We also demonstrate that signals promoting epicardial cell EMT are deficient in *EHC* and *Myh10∆* mutant embryos, and that migration of EPDCs into the myocardium is impaired. Together, these data indicate that the coronary defects observed in the *EHC* embryos are underpinned by compromised epicardial function, and suggest that NMHC IIB plays a crucial role in normal epicardial biology. Confirmation of our findings is provided from a recent study demonstrating that mice with an epicardial-specific knock out of *Myh10* display defects in coronary vessel development [53]. *Myh10* is a pleiotropic gene that performs multiple roles in different developmental processes including tension generation [78], growth factor receptor internalisation [79], cell adhesion [20], and extracellular matrix protein secretion [80]. Moreover, the different NMHC II isoforms have functionally distinct roles [24]. Further investigation of the molecular functions of NMHC IIB in the epicardium and other cardiac cell types may inform therapeutic strategies to reactivate epicardial processes in injured cardiac tissue and enhance coronary vessel repair and regeneration.

## Materials and methods

### Ethics statement

Experiments using animals were performed in accordance with legislation in the UK Animals (Scientific Procedures) Act of 1986 (PPL 70/8858 to Graham Morrissey). Experiments were approved by the University of Manchester Animal Welfare and Ethical Review Body.

### Mouse strains and genotyping

The *l11Jus*27 mouse line was generated from a balancer chromosome mutagenesis screen. The two mutations carried in the *l11Jus*27 mouse line were maintained *in trans* to 129S5.Inv(11)8Brd^Trp53-Wnt3^ [19]. Genomic DNA was prepared from ear punches of adult mice and yolk sacs (<E11.5) or tails (≥E11.5) of embryos. Genotypes were determined by PCR analysis with microsatellite marker *D11MIT327*, *D11MIT35*, *D11MIT31* or *D11MIT322* to differentiate between C57BL/6 (mutant) and 129S5 (wild type) strains of mice. *Myh10*^*tm7Rsad*^ mice were obtained from the MMRRC, and were crossed to *Tg(Nes-cre)*^*Wme*^ mice to generate global deletion of *Myh10* exon 2. *Tg(Myh6-cre)*^*2182Mds/J*^ mice were bred to *Myh10*^*tm7Rsad*^ mice to generate cardiomyocyte-specific deletion of *Myh10* exon 2. Genotyping primers are listed in S1 Table. PCR products were sequenced to confirm specificity of genotyping PCR reactions.

### Meiotic mapping and NMHC IIB sequencing

For mutation mapping, mice were bred to 129S5 wild type mice. Animals inheriting the *l11Jus27* mutation but not the balancer chromosome were selected for further breeding. *l11Jus*27 mice without the balancer were crossed to 129S5 wild type animals and progeny examined for recombination events. Recombination events were identified using microsatellite and SNP polymorphic markers between C57BL/6 and 129S5 mouse strains (primer sequences in S1 Table). Recombinant mice were intercrossed to *l11Jus*27 mice and progeny analysed. An absence of homozygous C57BL/6 pups indicated that the mutation was present in the recombinant animal. A timed mating was performed to confirm the mutant phenotype. Recombinant mouse 363, which only carried the ‘*EHC*’ point mutation, was bred with 129S5.Inv(11)8Brd^Trp53-Wnt3^ to generate the *EHC* mouse line. The *EHC* point mutation was identified by genomic sequencing of all annotated *Myh10* exons (UCSC genome Browser mm9 assembly). PCR amplification was performed on each exon, products precipitated, and cycle sequenced using Big Dye v1.1 reaction mix (ABI) according to manufacturer’s instructions (primer sequences in S1 Table).

### Complementation assay

To confirm genomic deletion of *Myh10* exon 2, PCR was performed for primers flanking exon 2 (Fig 6) and products were sequenced to confirm specificity to *Myh10*. Matings of *Myh10*∆/+ and *EHC*/+ male and female adult mice were set up and pregnancies were allowed to proceed to term. Neonate litters were culled and tail biopsies from euthanised mice were used to genotype each animal for the *Myh10*∆ and *EHC* mutations. The observed genotypes were compared to expected Mendelian ratios and data sets were analysed using a Chi-squared test with 2 degrees of freedom (http://graphpad.com/quickcalcs/chisquared1.cfm).

### Embryo dissection and analysis

Mice were set up for timed matings and the morning of the vaginal plug was defined as day E0.5. Mice were sacrificed according to Home Office Schedule 1 methods. Embryos were then dissected from decidua at the desired time point and subsequently imaged in PBS using a Leica MZ6 microscope and DFC420 camera.

### Protein structure predictive modelling

The three dimensional structure of residues 1–815 of mouse NMHC IIB were predicted using homology modelling. The sequences of mouse NMHC IIB and chicken smooth muscle myosin were aligned using ClustalW [81], and structure predicted by Modeller [82] using the known chicken myosin structure (PDB id 1BR1) [83] as a template. The sequence identity between the two proteins was 83% over the aligned region. Twenty models were built, and the one with the best score was used for further analysis.

### SDS-PAGE and Western blotting

Protein was extracted from E11.5 embryos by homogenisation in RIPA buffer. Protein concentrations were determined according to the manufacturer’s protocol (Biorad Protein Assay). Protein lysate (30 μg) was loaded onto a 10% polyacrylamide gel and separated by electrophoresis before being transferred to a PVDF membrane. Membranes were blocked overnight in 5% milk and incubated in anti-nonmuscle myosin II primary antibodies (NMIIA, Covance, PRB-440P, NMIIB Covance, PRB-445P, NMIIC, Covance, PRB-444P; 1:1000) or anti-beta-actin HRP conjugate (Sigma, A3854; 1:200,000) for 1 hour at room temperature. Membranes were subsequently incubated in HRP conjugated donkey anti-rabbit secondary antibody (Santa Cruz, SC-2313; 1:1000) for 1 hour at room temperature. Protein detection was performed using the ECL Plus Western Blotting detection system (GE Healthcare) according to manufacturer’s instructions.

### Quantitative real-time PCR

RNA was prepared from E12.5 wild type, heterozygous and mutant embryos using Tri reagent (Sigma). RNA (5 μg) was treated with RNase-free DNase 1 (Promega) and cDNA generated using random primers (Promega) and Bioscript reverse transcriptase (Bioline). Primers for *Myh10* qPCR are listed in S1 Table.

### Histology

Embryos were fixed in Bouin’s fixative or 4% paraformaldehyde (PFA), dehydrated and then cleared in xylene or histoclear. Embryos were embedded in paraffin and sectioned at 7 μm. After sectioning paraffin was removed from sections by washing 2 x 10 minutes in xylene or histoclear. Sections were then rehydrated and stained in haemotoxylin and eosin or Alcian blue and/or nuclear fast red before being mounted in depex mounting medium for analysis.

### Immunohistochemistry and immunofluorescence

Embryos and embryonic hearts were fixed overnight in 4% PFA and stored in 70% ethanol. For immunohistochemistry, whole hearts were incubated with primary antibodies anti-PECAM-1 (BD Biosciences, 550274; 1:100) for 1 hour at room temperature. For immunohistochemistry on tissue sections, samples were incubated in anti-smooth muscle alpha actin (Sigma, A5228; 1:400) for 1 hour at room temperature. Staining was developed using the Vectastain Elite ABC Kit (Vector, PK-6100) and visualised using the DAB substrate kit (Vector, SK-4100). For immunofluorescence on tissue sections hearts were dehydrated, embedded in paraffin and sectioned at 7 μm. Tissue sections were subjected to antigen retrieval by heating in citrate buffer (DAKO, S1699) as per the manufacturer’s instructions. Tissue was blocked prior to incubation in primary antibodies with either 10% (v/v) serum in PBS, or DAKO serum free protein block (DAKO, X0909). Heart sections were stained with primary antibodies directed against the following proteins: laminin (custom-made antibody, kind gift from Ray Boot-Handford, University of Manchester; 1:400); fibronectin (Santa Cruz, SC-6952; 1:50 dilution), collagen 1 (Gentaur, OARA02579; 1:400), fibronectin (Santa Cruz, sc-6952, 1:50), p65 (Santa Cruz, SC-372; 1:200), cardiac Troponin T (Abcam, ab106076; 1:400), NMHC IIB (Eurogentec, PRB-445P-050; 1:400), Raldh2 (Abcam, ab75674: 1:200), SM22α (Abcam, ab14106; 1:250), endomucin (Santa Cruz, SC-65495; 1:50), sarcomeric alpha-actinin (Sigma, EA-53; 1:500), and beta-catenin (Sigma, C2206, 1:250) overnight at 4° C. Embryos were incubated in species-specific fluorescent secondary antibodies (Jackson Immunochemicals or Invitrogen; 1:500 dilution) and slides mounted in DAPI mounting media (Vector).

### Blood cell peroxidase staining

Whole hearts were fixed for two hours at room temperature in 4% PFA. Hearts were washed 2X5 min in PBS, prior to incubation in the DAB substrate kit with added Nickel solution (Vector, SK-4100) according to manufacturer’s instructions.

### Cryo-immunohistochemistry

E14.5 embryonic hearts were dissected into ice cold PBS, embedded in OCT (R.A. Lamb) and snap frozen in liquid nitrogen. Cryosections (14 μm thick) were fixed in 4% PFA for 15 minutes, permeabilised in PBS + 0.1% (v/v) Triton X-100 for 15 minutes and subsequently blocked with PBS + 1% (w/v) BSA + 10% (v/v) normal goat or horse serum (Vector) for 1 hour. Sections were then incubated with the following primary antibodies diluted in PBS + 0.1% (v/v) Triton X-100 for 24 hours at 4°C: rabbit polyclonal anti-mouse Wt-1 (Calbiochem, CA1026, 1:300), goat anti-Snail (Abcam, ab53519, 1:100), rabbit anti-PHH3 (Millipore, 06–570, 1:300), anti-vimentin (Proteintech, 10366-1-AP, 1:50). Sections were then incubated with species-specific biotinylated secondary antibodies (Vector) at 1:500, for 2 hours, or FITC conjugated goat anti-rabbit secondary antibody (Sigma, F9887, 1:160) (detection of PHH3). Sections were incubated with Cy3 conjugated streptavidin (GE Healthcare, PA43001), diluted 1:3000 or 1:1000 (detection of Snail) for 30 minutes. Coverslips were mounted with Vectashield with DAPI (Vector, H-1200) and sealed with nail varnish. Slides were stored in the dark at 4°C and imaged within 48 hours.

### Epicardial cell culture

Cardiac fibroblast and cardiomyocyte cell populations were generated from E15.5 embryonic hearts according to previously described protocols [38, 39]. For the generation of epicardial cell cultures, embryonic hearts were dissected from E10.5–12.5 embryos and the atria and outflow tract were removed. Ventricular tissue was then carefully dissected into four pieces of comparable size, and each piece was placed onto a coverslip pre-coated with 0.1% gelatin (Sigma, G2500) (1 hour incubation at 37°C) in a 24 well tissue culture plate (Corning). Explants were cultured in 500μl DMEM (Sigma, D5796) supplemented with 15% (v/v) heat inactivated FBS (Gibco, 10500064) and 1% (v/v) Penicillin/Streptomycin (Sigma, P0781). Explants were incubated at 37°C with 5% CO_2_ and the media was replaced every 3 days until the cultures were required for experiments.

### Immunocytochemistry

Epicardial cells cultured for 72 hours were washed with tissue culture grade PBS + MgCl_2_ and CaCl_2_ (Sigma, D8662) and then fixed in 4% PFA for 10 minutes on an orbital shaker. The coverslips were then washed and cell monolayers were permeabilised by incubating in PBS + 0.1% (v/v) Triton X-100 for 15 minutes. Cultures were then blocked in either PBS + 10% (v/v) goat serum, or PBS + 1% (w/v) BSA, for at least 1 hour before addition of relevant primary antibody diluted in PBS + 0.1% (v/v) Triton X-100 for 1 hour: rabbit anti-ZO-1 (Invitrogen, 40–2300, 1:100), rabbit anti-Wt1 (1:300, SantaCruz, SC-192), goat anti-fibronectin (Santa Cruz, sc-6952, 1:100) or rabbit anti-NMHC IIB (Biolegend, PRB445P, 1:500). Unbound antibody was removed by washing the cultures with PBS before addition of appropriate biotinylated secondary antibody (Vector) diluted 1:500 in PBS + 0.1% (v/v) Triton X-100 for 1 hour. For visualisation of fibronectin, cultures were incubated with FITC conjugated secondary antibody (Sigma, F9887, 1:160) or developed using the Vectastain Elite ABC Kit (Vector, PK-6100) and visualised using the DAB substrate kit (Vector, SK-4100) as per manufacturer’s instructions. Cultures were washed and then incubated in Cy5 conjugated streptavidin (GE Healthcare, PA45001) diluted 1:500 in PBS + 0.1% (v/v) Triton X-100 for 30 minutes. Coverslips were washed and then treated with 100nM rhodamine-phalloidin (Cytoskeleton, PHDR1) for 30 minutes to allow visualisation of the actin cytoskeleton. Following a final wash, coverslips were mounted onto microscope slides using Vectashield with DAPI mounting media (Vector, H-1200), and stored at 4°C in the dark until imaging within 48 hours. The same protocol was employed for immunofluorescence staining of NMHC IIB in cultured cardiac fibroblasts and myocytes populations on gelatin coated glass coverslips.

### TUNEL assay

Paraffin sections were rehydrated and subjected to TUNEL staining (Promega DeadEnd Fluorometric TUNEL System, G3250) as per the manufacturer’s instructions.

### Fluorescent microscopy image collection and analysis

Images were collected on an Olympus BX51 upright microscope and captured using a Coolsnap ES camera (Photometrics) through MetaVue Software (Molecular Devices). Specific band pass filter sets for DAPI, FITC, Cy3 and Cy5 were used to prevent bleed through from one channel to the next. Images were then processed and analysed using ImageJ software (Wayne Rasband, NIH, USA). (http://rsb.info.nih.gov/ij). IMARIS (Bitplane) 7.3.4 software was used to analyse the fluorescence intensity in epicardial and myocardial regions for ECM and p65 quantification. For each individual cell in the epicardial and myocardial layers, mean fluorescence intensities (MFI), Alexa488, and Alexa 647 were measured in pixels. Twenty randomly selected areas of the epicardium and myocardium were measured per tissue section. Five sections per embryo, and three embryos of each genotype, were measured. Myocardial measurements were taken from the compact myocardium as defined by morphology rather than trabeculae or endocardium. Two-tailed student t-test was used to test the significance of differences between two sets of data. Further details of data analysis techniques, statistical tests and numbers of samples used are provided in figure legends.

### Scratch wound assay

Epicardial cells were cultured from ventricular explants as described above. After 48 hours in culture, explants were removed and the cell monolayer was briefly washed twice with complete media and returned to the incubator for 24 hours. Cell monolayers were scratched using a P10 pipette tip, rinsed twice in complete media, and photographed at 10 minute intervals for 20 hours using an AS MDW live cell imaging system, maintained at 37°C and 5% CO_2_. Ten cells at the leading edge of the denuded area were tracked per field of view, using MTrackJ in ImageJ. Total tracked cells = 240 control and 270 mutant. Cultures were generated from at least four hearts for each genotype. Data sets for directional persistence and migration speed measurements were subjected to Mann-Whitney U test to assess statistical significance.

### Whole mount *in situ* hybridisation

Embryos were fixed overnight in 4% PFA and *in situ* hybridisation was performed as previously described [84, 85]. *Tbx18* and *Shox2* plasmids were obtained from A. Kispert (Institute of Molecular Biology Hannover, Germany). Anti-sense probes were synthesized from linear template DNA using RNA polymerase, digoxygenin nucleotide mix and transcription buffer (Roche). RNA probes were purified by precipitation and added to hybridisation mix at a concentration of 0.5 μg/μl.

### Transmission electron microscopy

Samples were prepared for electron microscopy as described previously [86]. Sections (50–70 nm-thick) were generated using a Reichert-Jung Ultracut (Leica Microsystems, UK) and stained using 2% uranyl acetate and 0.3% lead citrate. Sections were examined with an FEI Tecnai 12 Biotwin transmission electron microscope. Images were recorded using a Gatan Orius SC1000 camera (11 Mpixels, 4008 x 2672).

## Supporting information

S1 Fig *Myh10*∆ homozygous mutants do not synthesise full-length NMHC IIB protein and exhibit embryonic lethality. A: Western blot analysis of E11.5 wild type, *Myh10*∆ heterozygous control and *Myh10*∆ homozygous mutant heart protein extracts for NMHC IIB expression using a C-terminal antibody. NMHC IIB is abundant in controls but not detected in homozygous mutant samples. B: Table showing expected Mendelian frequencies vs observed frequencies of wild type, *Myh10*∆ heterozygous and *Myh10*∆ homozygous mutant progeny from intercrossed *Myh10*∆ heterozygous animals (Chi squared test, p = 0.0035). C: Table showing expected Mendelian frequencies vs observed frequencies of wild type, *EHC*/+ or *Myh10*∆/+ heterozygous and *EHC*/*Myh10*∆ compound heterozygous mutant progeny from complementation test of intercrossed *Myh10*∆/+ and *EHC*/+ heterozygous animals (Chi squared test, p = 0.0278). The *Myh10*∆ allele does not rescue the *EHC* embryonic lethality phenotype, and therefore fails to complement the *EHC* allele.
(TIFF)

S2 Fig Analysis of cardiac cell morphology in *EHC* mutants. Comparison of ventricular cells in heterozygous control embryos at E12.5 (A) and *EHC* mutant hearts (B) at E12.5 reveals mutant cells have large nuclei (B, arrows), consistent with prior descriptions of cytokinesis defects in *Myh10* knockout embryos. Immunofluorescent staining at E10.5 of heterozygous control (C) and *EHC* mutant hearts (D). Cell-cell contacts are stained with β-catenin (green) and myofibrils are stained with α-actinin (red), DAPI labels the nuclei (blue). Large nuclei are also present in *EHC* mutant embryos at E10.5 (D, asterisks). EM imaging shows epicardial cells in control embryos (E) and *EHC* mutant embryos (F-G). Multiple epicardial cells in *EHC* mutants display abnormal morphology (F-G, white arrows), and large nuclei are present in cells within the underlying myocardial tissue (F, black arrow). The percentage of epicardial cells with abnormal morphology compared to total epicardial cell number was calculated from images of 5 individual control embryos and 4 individual *EHC* mutant embryos. Overall there was a statistically significant difference in the percentage of abnormal epicardial cells in control embryos (11% abnormal) as compared to *EHC* mutants (71% abnormal; Fishers exact text p<0.0001). Alcian blue staining indicates the presence of glycosaminoglycans in endocardial cushion tissue in control littermate (H, J) and *EHC* mutant heart (I, K). The areas magnified in panels J and K are indicated with boxes on panels H and I. Scale bars: A-D: 25 μm, E-F: 5 μm, G = 17 μm, H-I: 1mm.
(TIF)

S3 Fig Analysis of coronary vessel endothelial and smooth muscle cells in *EHC*/*Myh10*∆ and *Myh10*∆ homozygous mutants. PECAM-1 staining at E16.5 reveals prominent vessels on the dorsal surface of the *Myh10∆/+* control heart (A). The *Myh10∆/∆* (B) and *Myh10∆/EHC* (C) mutant hearts do not have surface vessels, but rather clusters of PECAM-1 immunoreactive cells (arrows). Section immunohistochemistry for SMαA on D: *EHC* heterozygote, E: *EHC* homozygous mutant, or F: *EHC/Myh10*∆ compound heterozygote at E16.5. Higher magnifications of the boxed areas in sections D-F reveals vessels ringed with SMαA cells in G: *EHC* heterozygote, but no such structures are present in H: *EHC* homozygous mutant or I: *EHC/Myh10*∆ compound heterozygote. Similar results were observed for *Myh10*∆ homozygous mutant embryos. J: High magnification view of PECAM-1 immunostaining on control heart at E16.5 reveals large coronary vessels. K: *EHC* mutant heart at E16.5 immunostained for PECAM-1 shows some evidence of a capillary network, along with surface blisters, but no large vessels. Scale bars: A-C = 1mm, D-F and J-K = 0.25mm; G-I = 0.05mm. Abbreviations: PECAM: platelet endothelial cell adhesion molecule-1, SMαA: smooth muscle alpha-actin, ∆: *Myh10*∆.
(TIF)

S4 Fig Analysis of coronary vessel development in *Myh10* cardiomyocyte-specific knock out embryos. A-B: Ventral and dorsal views, respectively, of control littermate showing prominent blood-filled coronary vessels (arrows) at dissection at E18.5. C-D: Ventral and dorsal views, respectively, of *Myh10* cardiomyocyte-specific knock out heart showing prominent blood-filled coronary vessels (arrows) at dissection at E18.5. E: PECAM-1 staining reveals prominent vessels (arrows) on the surface of the control heart at E16.5. F: PECAM-1 staining reveals prominent vessels (arrows) on the surface of the *Myh10* cardiomyocyte-specific knock out heart at E16.5. Scale bars: 1 mm (A-F). Each pair of images is taken at the same magnification. Genotypes are labeled on the image. Abbreviations: *α-MHC*: α-myosin heavy chain encoded by *Myh6*, PECAM: platelet endothelial cell adhesion molecule-1.
(TIF)

S5 Fig The atrioventricular valves are present in *EHC* mutant hearts. Nuclear fast red staining of E14.5 (A-B) and E16.5 (C-D) *EHC* heterozygote control and *EHC* homozygous mutant hearts demonstrating the presence of both the mitral and tricuspid atrioventricular valve leaflets (arrows). Scale bars: 1 mm.
(TIF)

S6 Fig Analysis of fibronectin distribution in epicardial explant cultures. Immunohistochemistry on epicardial explant cultures for control (A) and *Myh10∆* homozygous mutant (E) to detect fibronectin (arrows). Higher power images show a well-organised fibronectin fibril network in control sample (B, arrows), but not in *Myh10∆* homozygous mutant (F). Immunofluorescence staining confirms that control samples have prominent localization of fibronectin (C, green) while staining levels are reduced in mutants (G). Nuclei are visualised with DAPI (blue). The underlying ECM in the explant culture dish was assayed for the presence of fibronectin following removal of explant cells with 20mM ammonium hydroxide, revealing greater prominence of fibronectin in the control sample (D, arrows) as compared to the *Myh10∆* homozygous mutant sample (H, arrows). Explants were cultured on gelatin-coated plates. Scale bars: A and E = 100 μm, B-C and F-G = 200 μm.
(TIF)

S7 Fig Analysis of cell proliferation in control and *Myh10∆/∆* homozygous mutants at E9.5. Phosphohistone H3 immunofluorescence (green) labels proliferating cells (arrows) in control (A) and *Myh10∆* homozygous mutant (B) cardiac sagittal sections at E9.5. No statistically significant difference (C) was detected in the percentage of proliferating cells in mutant samples as compared to controls, (two-tailed t-test. p = 0.57). Three sections each from three embryos of each genotype were counted. PHH3 stained cells in cardiac tissue only (identified by morphology) were counted. DAPI positive nuclei (blue) in cardiac tissue only were counted to determine total cell number in each sample. Abbreviations: PHH3: phosphohistone H3, *∆/∆* = *Myh10∆*homozygous mutant. Scale bars: 50 μm.
(TIF)

S1 Movie Movie of control epicardium scratch wound assay. Time-lapse microscopy imaging of wound healing in control (+/+) epicardial cell cultures. A ‘denuded’ area was created by scratching D3 epicardial cell culture monolayers with a P10 pipette tip. Point visiting was used to image the same field of view at 10-minute intervals for a period of 20 hours. Ten cells per field of view were tracked over the duration of the experiment using the MTrackJ plugin in ImageJ (as indicated by multicoloured lines). Lines indicate the tracking of cells that were measured for speed and directional persistence.
(AVI)

S2 Movie Movie of *Myh10∆/∆* mutant epicardium scratch wound assay. Lines indicate the tracking of cells that were measured for speed and directional persistence, using the same methodology as reported for S1 Movie.
(AVI)

S1 Table Primer sequences used for genotyping and sequencing. (DOCX)
