# Supplementary figures and images for: Non-muscle myosin IIB (Myh10) is required for epicardial function and coronary vessel formation during mammalian development
Source: PLoS Genet. 2017 Oct 30;13(10):e1007068. doi: 10.1371/journal.pgen.1007068 (PMC5697871; doi:10.1371/journal.pgen.1007068)

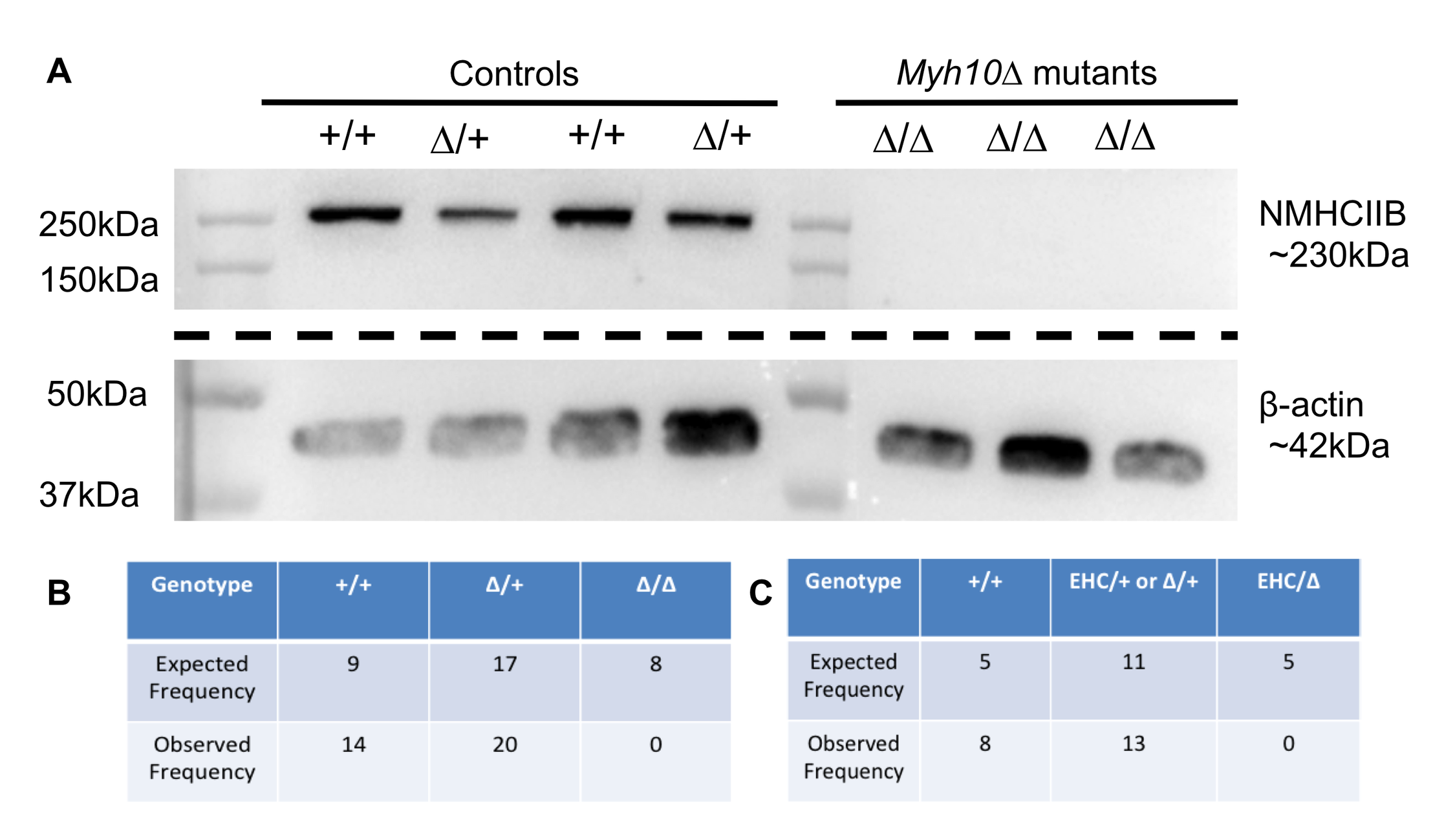

Supplement: S1 Fig — A: Western blot analysis of E11.5 wild type, Myh10∆ heterozygous control and Myh10∆ homozygous mutant heart protein extracts for NMHC IIB expression using a C-terminal antibody. NMHC IIB is abundant in controls but not detected in homozygous mutant samples. B: Table showing expected Mendelian frequencies vs observed frequencies of wild type, Myh10∆ heterozygous and Myh10∆ homozygous mutant progeny from intercrossed Myh10∆ heterozygous animals (Chi squared test, p = 0.0035). C: Table showing expected Mendelian frequencies vs observed frequencies of wild type, EHC/+ or Myh10∆/+ heterozygous and EHC/Myh10∆ compound heterozygous mutant progeny from complementation test of intercrossed Myh10∆/+ and EHC/+ heterozygous animals (Chi squared test, p = 0.0278). The Myh10∆ allele does not rescue the EHC embryonic lethality phenotype, and therefore fails to complement the EHC allele. (TIFF) [file pgen.1007068.s001.tiff]

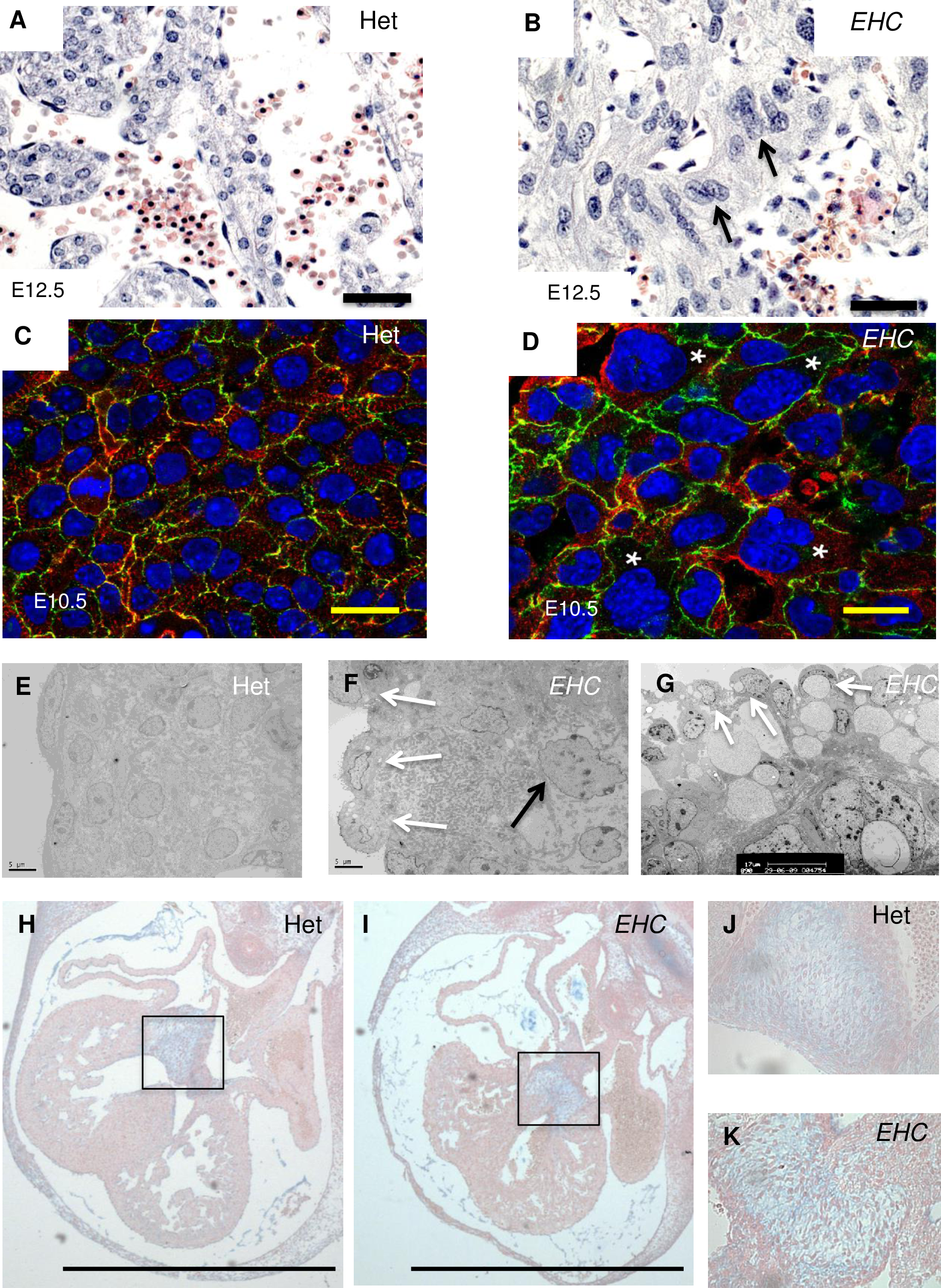

Supplement: S2 Fig — Comparison of ventricular cells in heterozygous control embryos at E12.5 (A) and EHC mutant hearts (B) at E12.5 reveals mutant cells have large nuclei (B, arrows), consistent with prior descriptions of cytokinesis defects in Myh10 knockout embryos. Immunofluorescent staining at E10.5 of heterozygous control (C) and EHC mutant hearts (D). Cell-cell contacts are stained with β-catenin (green) and myofibrils are stained with α-actinin (red), DAPI labels the nuclei (blue). Large nuclei are also present in EHC mutant embryos at E10.5 (D, asterisks). EM imaging shows epicardial cells in control embryos (E) and EHC mutant embryos (F-G). Multiple epicardial cells in EHC mutants display abnormal morphology (F-G, white arrows), and large nuclei are present in cells within the underlying myocardial tissue (F, black arrow). The percentage of epicardial cells with abnormal morphology compared to total epicardial cell number was calculated from images of 5 individual control embryos and 4 individual EHC mutant embryos. Overall there was a statistically significant difference in the percentage of abnormal epicardial cells in control embryos (11% abnormal) as compared to EHC mutants (71% abnormal; Fishers exact text p<0.0001). Alcian blue staining indicates the presence of glycosaminoglycans in endocardial cushion tissue in control littermate (H, J) and EHC mutant heart (I, K). The areas magnified in panels J and K are indicated with boxes on panels H and I. Scale bars: A-D: 25 μm, E-F: 5 μm, G = 17 μm, H-I: 1mm. (TIF) [file pgen.1007068.s002.tif]

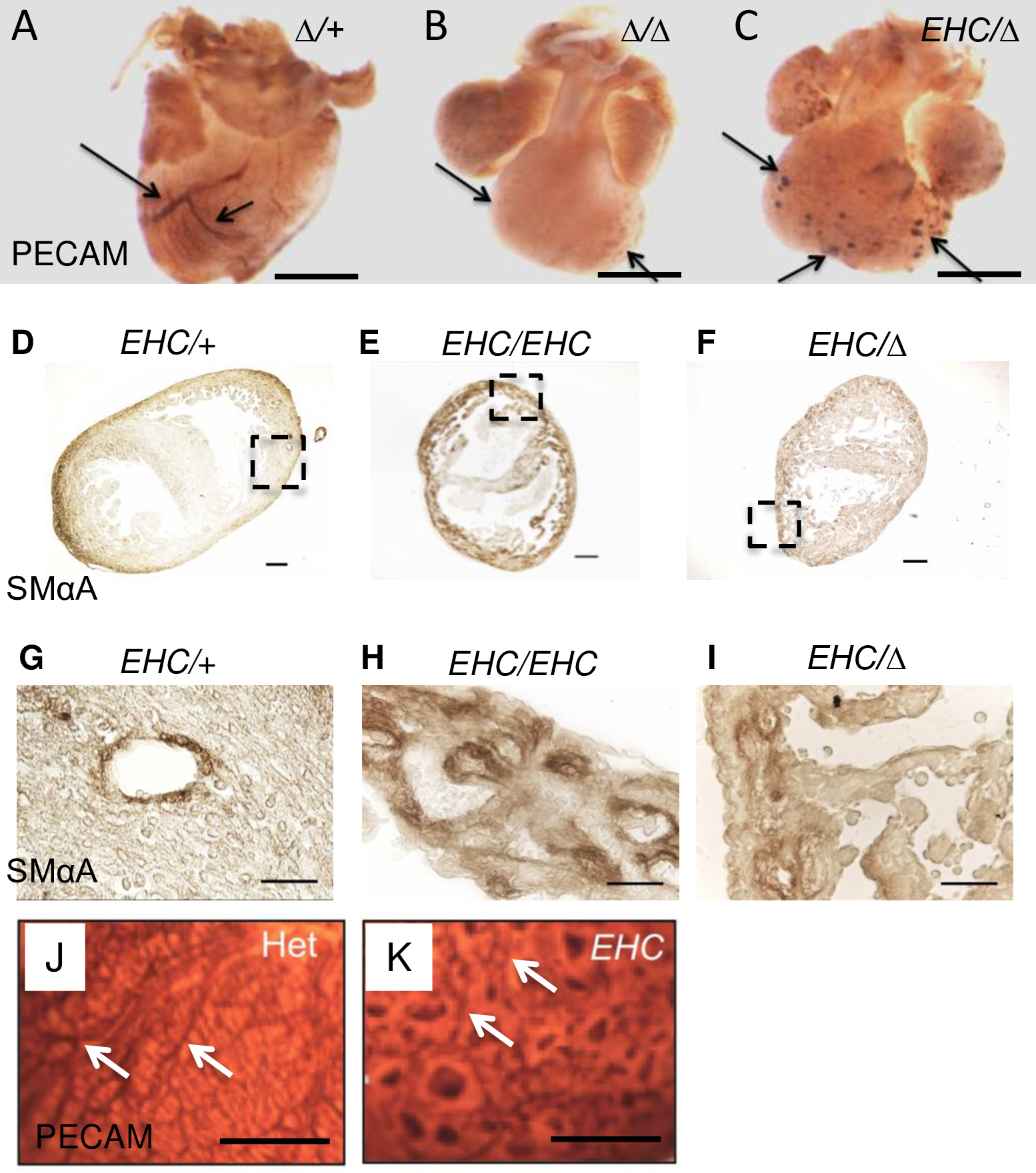

Supplement: S3 Fig — PECAM-1 staining at E16.5 reveals prominent vessels on the dorsal surface of the Myh10∆/+ control heart (A). The Myh10∆/∆ (B) and Myh10∆/EHC (C) mutant hearts do not have surface vessels, but rather clusters of PECAM-1 immunoreactive cells (arrows). Section immunohistochemistry for SMαA on D: EHC heterozygote, E: EHC homozygous mutant, or F: EHC/Myh10∆ compound heterozygote at E16.5. Higher magnifications of the boxed areas in sections D-F reveals vessels ringed with SMαA cells in G: EHC heterozygote, but no such structures are present in H: EHC homozygous mutant or I: EHC/Myh10∆ compound heterozygote. Similar results were observed for Myh10∆ homozygous mutant embryos. J: High magnification view of PECAM-1 immunostaining on control heart at E16.5 reveals large coronary vessels. K: EHC mutant heart at E16.5 immunostained for PECAM-1 shows some evidence of a capillary network, along with surface blisters, but no large vessels. Scale bars: A-C = 1mm, D-F and J-K = 0.25mm; G-I = 0.05mm. Abbreviations: PECAM: platelet endothelial cell adhesion molecule-1, SMαA: smooth muscle alpha-actin, ∆: Myh10∆. (TIF) [file pgen.1007068.s003.tif]

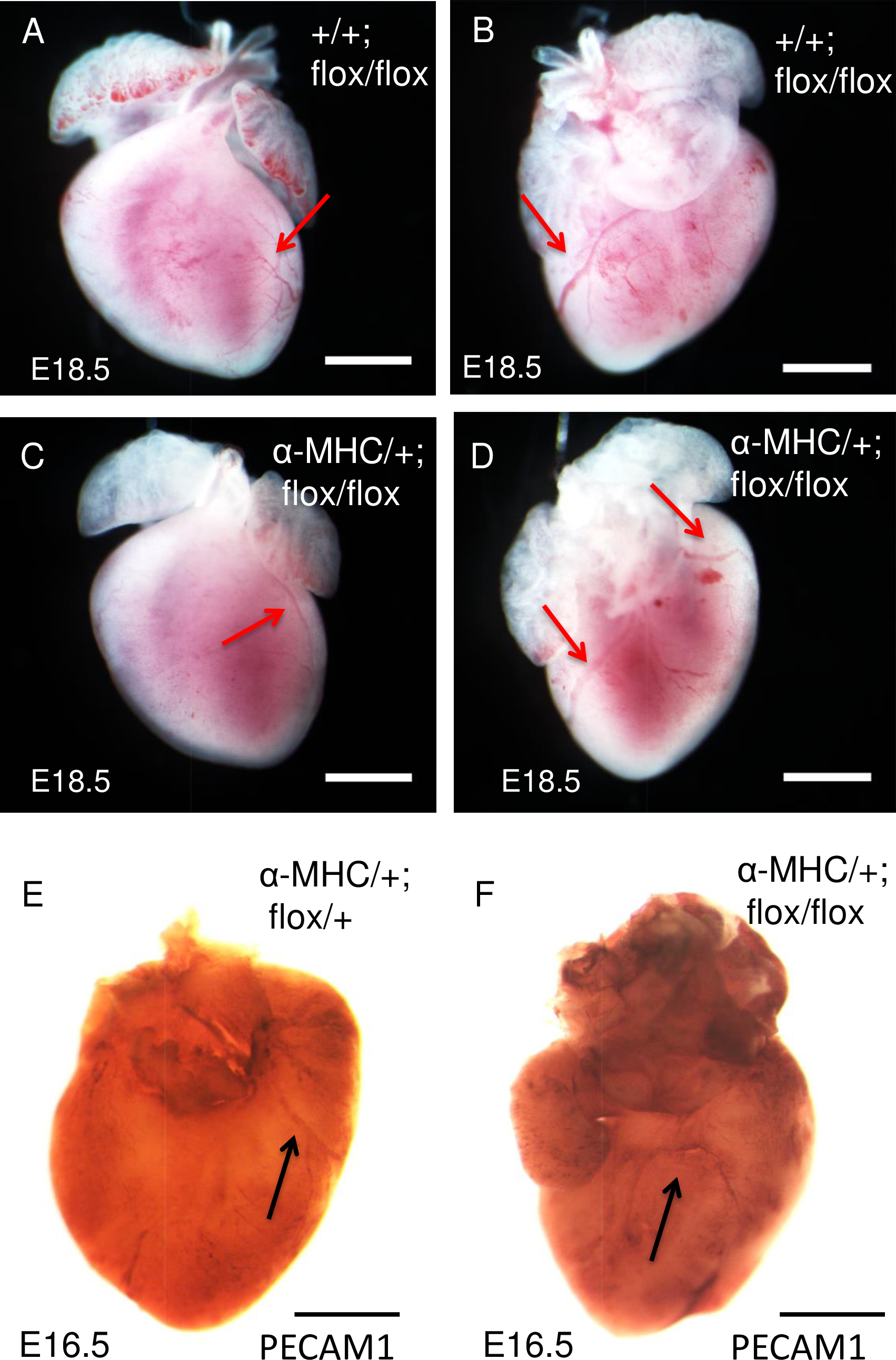

Supplement: S4 Fig — A-B: Ventral and dorsal views, respectively, of control littermate showing prominent blood-filled coronary vessels (arrows) at dissection at E18.5. C-D: Ventral and dorsal views, respectively, of Myh10 cardiomyocyte-specific knock out heart showing prominent blood-filled coronary vessels (arrows) at dissection at E18.5. E: PECAM-1 staining reveals prominent vessels (arrows) on the surface of the control heart at E16.5. F: PECAM-1 staining reveals prominent vessels (arrows) on the surface of the Myh10 cardiomyocyte-specific knock out heart at E16.5. Scale bars: 1 mm (A-F). Each pair of images is taken at the same magnification. Genotypes are labeled on the image. Abbreviations: α-MHC: α-myosin heavy chain encoded by Myh6, PECAM: platelet endothelial cell adhesion molecule-1. (TIF) [file pgen.1007068.s004.tif]

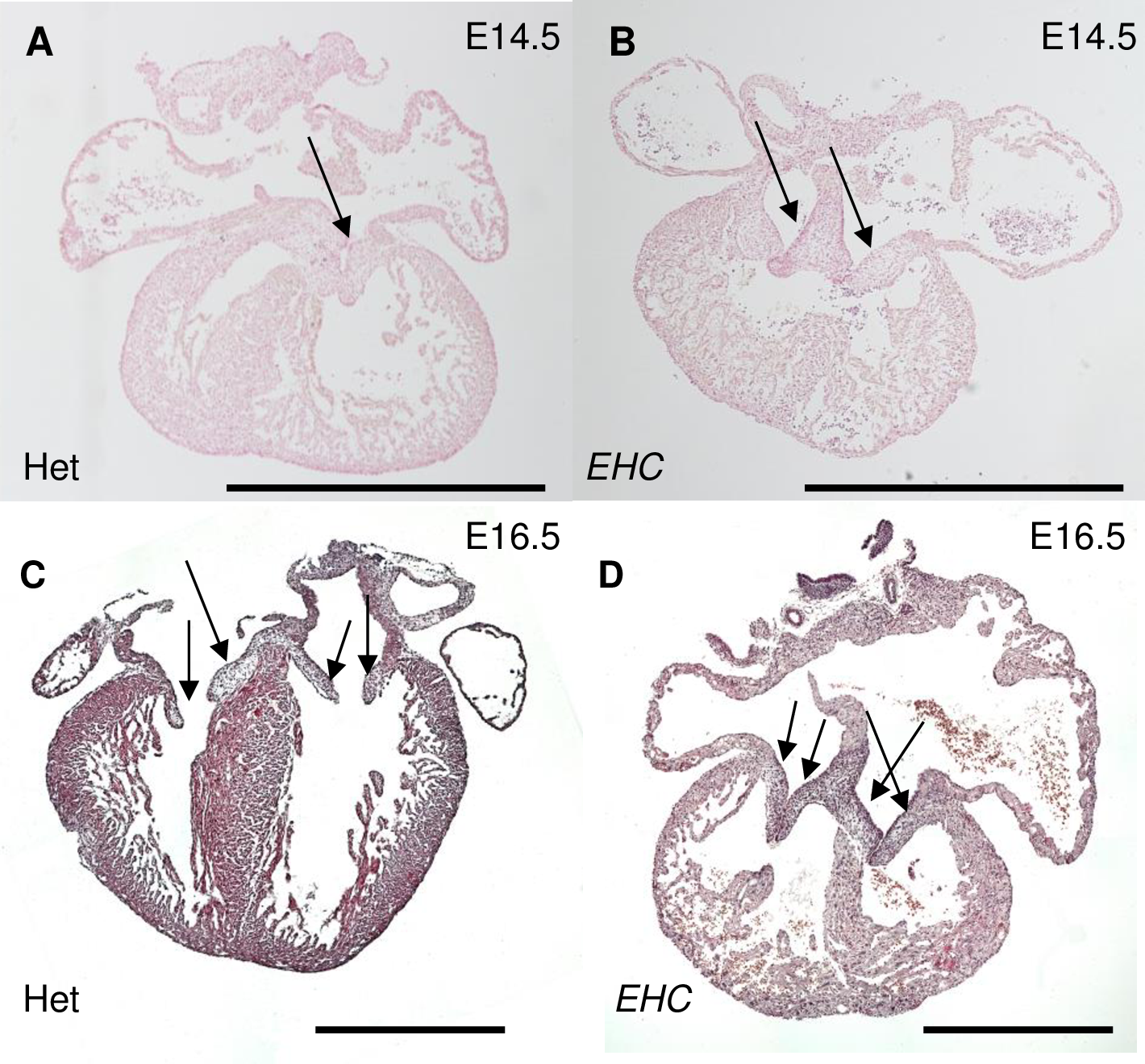

Supplement: S5 Fig — Nuclear fast red staining of E14.5 (A-B) and E16.5 (C-D) EHC heterozygote control and EHC homozygous mutant hearts demonstrating the presence of both the mitral and tricuspid atrioventricular valve leaflets (arrows). Scale bars: 1 mm. (TIF) [file pgen.1007068.s005.tif]

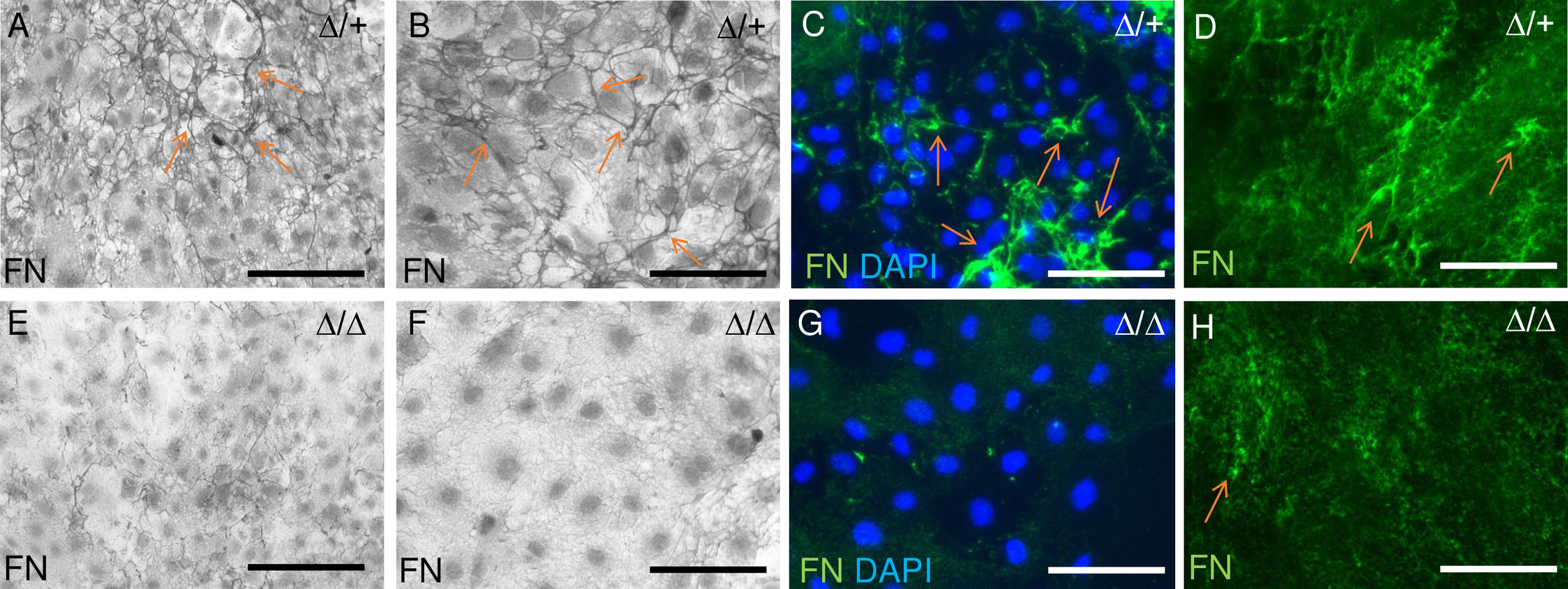

Supplement: S6 Fig — Immunohistochemistry on epicardial explant cultures for control (A) and Myh10∆ homozygous mutant (E) to detect fibronectin (arrows). Higher power images show a well-organised fibronectin fibril network in control sample (B, arrows), but not in Myh10∆ homozygous mutant (F). Immunofluorescence staining confirms that control samples have prominent localization of fibronectin (C, green) while staining levels are reduced in mutants (G). Nuclei are visualised with DAPI (blue). The underlying ECM in the explant culture dish was assayed for the presence of fibronectin following removal of explant cells with 20mM ammonium hydroxide, revealing greater prominence of fibronectin in the control sample (D, arrows) as compared to the Myh10∆ homozygous mutant sample (H, arrows). Explants were cultured on gelatin-coated plates. Scale bars: A and E = 100 μm, B-C and F-G = 200 μm. (TIF) [file pgen.1007068.s006.tif]

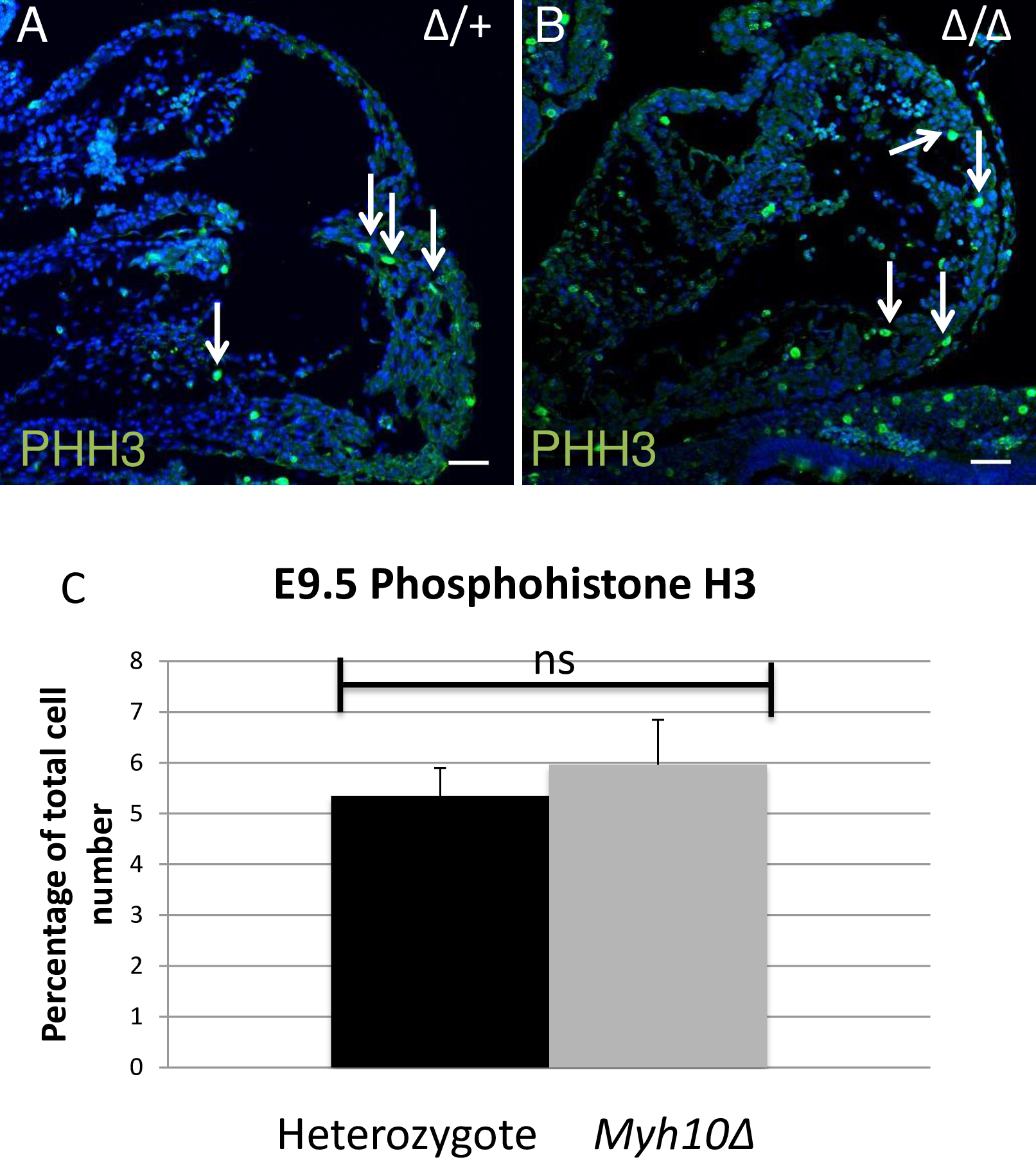

Supplement: S7 Fig — Phosphohistone H3 immunofluorescence (green) labels proliferating cells (arrows) in control (A) and Myh10∆ homozygous mutant (B) cardiac sagittal sections at E9.5. No statistically significant difference (C) was detected in the percentage of proliferating cells in mutant samples as compared to controls, (two-tailed t-test. p = 0.57). Three sections each from three embryos of each genotype were counted. PHH3 stained cells in cardiac tissue only (identified by morphology) were counted. DAPI positive nuclei (blue) in cardiac tissue only were counted to determine total cell number in each sample. Abbreviations: PHH3: phosphohistone H3, ∆/∆ = Myh10∆homozygous mutant. Scale bars: 50 μm. (TIF) [file pgen.1007068.s007.tif]
