# Supplementary material for: Non-muscle myosin IIB (Myh10) is required for epicardial function and coronary vessel formation during mammalian development
Source: PLoS Genet. 2017 Oct 30;13(10):e1007068. doi: 10.1371/journal.pgen.1007068 (PMC5697871; doi:10.1371/journal.pgen.1007068)
Supplement: S1 Table — (DOCX) [file pgen.1007068.s010.docx]

Supplemental File 8. Primer sequences used for genotyping and sequencing.

| Reaction | Forward sequence | Reverse sequence |
| --- | --- | --- |
| D11MIT327 | ATTACAGTTGACTGATACCAATCAGC | TCAGGCTCCACTGTGAAA TG |
| D11MIT35 | AGTAACATGGAACATCGACGG | TGCTCAGCTCTGGAGTGCTA |
| D11MIT31 | \|  \| \| --- \|   GCCTGAATTCACATGGTGG | AGAATAAGTAAACCCAGCTGCG |
| D11MIT322 | TGTCCTGCATACCCCTTTG | GATTAAGTACAGGCAACTGCAGG |
| D11MIT4 | CAGTGGGTCATCAGTACAGCA | AAGCCAGCCCAGTCTTCATA |
| Myh10 point mutation | TGCTAGATCAGTAGGCTGTGC | ATCCAGATGTAGTGGTGCATG |
| Myh10 delta exon 2 genotyping | GGTCATCCAGAACTGTGTTGT | TGGTCTTCTACTCTTCTTTGC |
| Myh10 flox allele (non-deleted) | CTGACATGCTTGAACGAAGC | CAGAGGCATGCAGATCTCTGT |
| Cre | GAACCTGATGGACATGTTCAGG | AGTGCGTTCGAACGCTAGAGCCTGT |
| Cre internal control (Myogenin) | TTACGTCCATCGTGGACAGC | TGGGCTGGGTGTTAGCCTTA |
| Myh10 qPCR | GGAAGAAACGCCATGAGATGC | GTGCATAGAATTGACTGGTCC |
